# Mechanistic Origin of Superionic Lithium Diffusion in Anion-Disordered Li_6_PS_5_*X* Argyrodites

**DOI:** 10.1021/acs.chemmater.0c03738

**Published:** 2021-03-03

**Authors:** Benjamin J. Morgan

**Affiliations:** † Department of Chemistry, University of Bath, Claverton Down, Bath BA2 7AY, U.K.; ‡ The Faraday Institution, Quad One, Harwell Science and Innovation Campus, Didcot OX11 0RA, U.K.

## Abstract

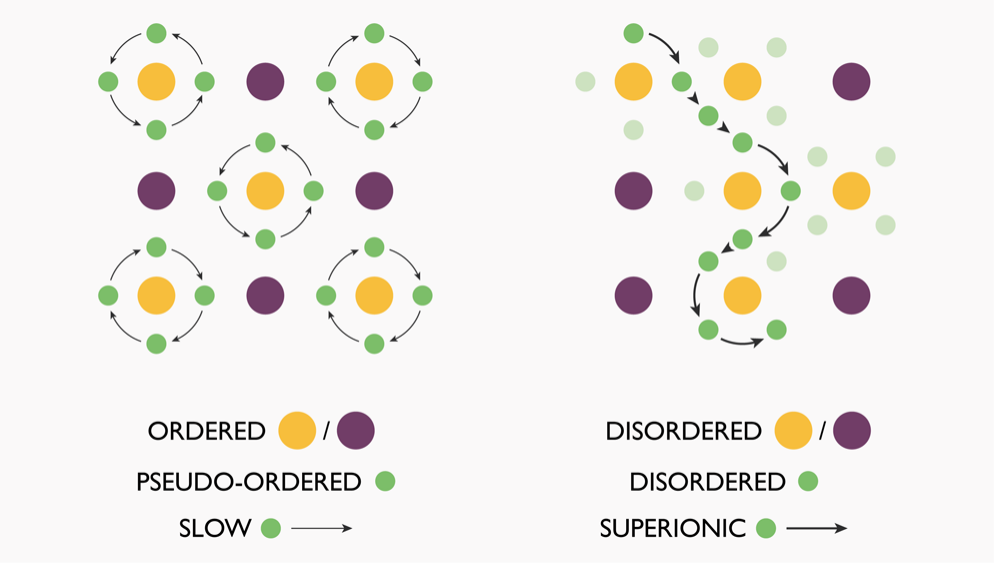

The rational development of fast-ion-conducting solid electrolytes for all-solid-state lithium-ion batteries requires understanding the key structural and chemical principles that give some materials their exceptional ionic conductivities. For the lithium argyrodites Li_6_PS_5_X (X = Cl, Br, or I), the choice of the halide, X, strongly affects the ionic conductivity, giving room-temperature ionic conductivities for X = {Cl,Br} that are ×10^3^ higher than for X = I. This variation has been attributed to differing degrees of S/X anion disorder. For X = {Cl,Br}, the S/X anions are substitutionally disordered, while for X = I, the anion substructure is fully ordered. To better understand the role of substitutional anion disorder in enabling fast lithium-ion transport, we have performed a first-principles molecular dynamics study of Li_6_PS_5_I and Li_6_PS_5_Cl with varying amounts of S/X anion-site disorder. By considering the S/X anions as a tetrahedrally close-packed substructure, we identify three partially occupied lithium sites that define a contiguous three-dimensional network of face-sharing tetrahedra. The active lithium-ion diffusion pathways within this network are found to depend on the S/X anion configuration. For anion-disordered systems, the active site–site pathways give a percolating three-dimensional diffusion network; whereas for anion-ordered systems, critical site–site pathways are inactive, giving a disconnected diffusion network with lithium motion restricted to local orbits around S positions. Analysis of the lithium substructure and dynamics in terms of the lithium coordination around each sulfur site highlights a mechanistic link between substitutional anion disorder and lithium disorder. In anion-ordered systems, the lithium ions are pseudo-ordered, with preferential 6-fold coordination of sulfur sites. Long-ranged lithium diffusion would disrupt this SLi_6_ pseudo-ordering, and is, therefore, disfavored. In anion-disordered systems, the pseudo-ordered 6-fold S–Li coordination is frustrated because of Li–Li Coulombic repulsion. Lithium positions become disordered, giving a range of S–Li coordination environments. Long-ranged lithium diffusion is now possible with no net change in S–Li coordination numbers. This gives rise to superionic lithium transport in the anion-disordered systems, effected by a concerted string-like diffusion mechanism.

## 1 Introduction

Lithium-ion-conducting solid electrolytes are considered candidate materials for use in future all-solid-state lithium-ion batteries.^1−3^ Present-day commercial lithium-ion batteries use liquid-organic electrolytes; these are flammable, raising safety issues, and have narrow electrochemical stability windows, preventing their use with energy-dense high-voltage electrodes. One possible solution is to instead use solid electrolytes, which ideally should be electrochemically inert, mechanically robust, have negligible electronic transport, and have high lithium-ion conductivities.^4^

Although a number of highly conducting solid lithium-ion electrolytes are known, none meet all the criteria for general commercial use.^1,4−6^ Identifying new solid lithium-ion electrolytes is an active area of research,^3^ with strategies ranging from targeted chemical modification of known solid electrolytes, to improve their conductivities,^7−11^ to high-throughput screening of new materials.^12−15^ In both cases, it is useful to understand why some materials are highly conducting, yet others are not.^3,16−20^ Such an understanding can help inform chemical strategies for optimizing the ionic conductivities of known materials, or can provide selection criteria for identifying new promising electrolytes. Particular insight can be gained from studying families of solid electrolytes that are superficially similar—such as those that share a common structural motif—but that exhibit quite different ionic conductivities,^2,19^ as this can help reveal the fundamental mechanisms and key material characteristics that govern fast-ion conduction.

One family of promising lithium-ion solid-electrolytes are the lithium argyrodites Li_6_PS_5_X (X = Cl, Br, or I).^11,21−25^ While Li_6_PS_5_Cl and Li_6_PS_5_Br exhibit high room-temperature ionic conductivities (σ_RT_ ≈ 10^–3^ S cm^–1^), Li_6_PS_5_I is considerably less conductive (σ_RT_ ≈ 10^–6^ S cm^–1^).^26,27^ The large difference between X = {Cl,Br} and X = I is notable because these three materials have topologically identical crystal structures, suggesting the same lithium-ion diffusion pathways should exist in each system. This inverse correlation between anion size and ionic conductivity also runs counter to the trend seen in other families of solid electrolytes, for example, thio-LISICON and NASICON, in which larger, more polarizable, less-electronegative anions are associated with increased ionic conductivities^2^—with this relationship often attributed to a combination of larger anions giving an increased accessible volume for the diffusing lithium ions and weaker lithium–anion electrostatic interactions.

A partial explanation for the ionic conductivity trend in the Li_6_PS_5_X argyrodites comes from the observation that in these materials, high conductivities are correlated with substitutional S/X anion disorder.^23,27^ In Li_6_PS_5_I, the anions are fully ordered, and S and I atoms fully occupy crystallographically distinct 4c and 4a Wyckoff positions, respectively. In Li_6_PS_5_Cl and Li_6_PS_5_Br, the S and Cl, or S and Br, atoms are substitutionally disordered, which has been attributed to their similar ionic radii^11,24,28^ giving a low formation energy for S/X antisites.^29,30^ Molecular dynamics simulations of Li_6_PS_5_X in which the degree of S/X disorder has been systematically varied provide additional evidence for a causal link between anion substitutional disorder and fast lithium-ion transport.^22,30−34^

Simulations performed on Li_6_PS_5_X models with fully ordered S/X atoms predict low lithium diffusion coefficients and highly localized lithium motion, with lithium ions restricted to discrete “cages” surrounding the S atoms. In contrast, simulations performed on S/X-disordered models predict high lithium diffusion coefficients, with lithium ions moving through a contiguous three-dimensional diffusion network. Despite this experimental and computational evidence linking lithium-ion conductivities in Li_6_PS_5_X argyrodites with the degree of S/X disorder, a mechanistic model that explains this relationship is currently lacking.

To address this question, we have performed a first-principles molecular dynamics study of Li_6_PS_5_I and Li_6_PS_5_Cl with varying amounts of S/X anion-site disorder. We find that the lithium substructure can be generally described in terms of partial occupation of three crystallographically distinct tetrahedral sites that define a contiguous three-dimensional network. The pattern of active and inactive lithium-ion diffusion paths within this network, however, depends on the degree of S/X disorder. In anion-ordered systems, lithium site positions are displaced toward neighboring sulfur sites because of electrostatic S–Li attraction, giving an ordered pattern of “inactive” site–site paths. In anion-disordered systems, however, the lithium site positions are statically disordered, and the set of active site–site paths forms a percolating three-dimensional network that permits long-ranged lithium diffusion.

We also have analyzed our simulation trajectories by considering clusters of lithium ions as “coordination polyhedra” located around S anions. This perspective provides insight into the spatial correlations and collective dynamics in these groups of lithium ions. In the anion-ordered systems, the lithium-ions are pseudo-ordered, and preferentially form 6-coordinate polyhedra around sulfur atoms. While lithium movement within these SLi_6_ units is frequent, lithium exchange between SLi_6_ units is rare on a simulation timescale. We explain this by considering lithium exchange as a form of “defect formation”, which is energetically disfavored. In the anion-disordered systems, however, strong Coulombic interactions between nearby lithium ions frustrate the otherwise preferable 6-fold S–Li coordination, producing a range of disordered SLi_*x*_ (*x* ≥ 6) coordination environments. Lithium movement between coordination polyhedra is now possible without a net change in S–Li coordination, making long-ranged lithium diffusion a viable low-energy process. Further analysis of the dynamical correlations between mobile lithium ions reveals a concerted string-like “superionic” diffusion mechanism in the anion-disordered argyrodites. These results provide a mechanistic explanation for the exceptional ionic conductivities of anion-disordered Li_6_PS_5_X argyrodites, and show how configurational framework disorder in solid electrolytes can cause static disorder amongst mobile ions, which consequently facilitates superionic conductivity.

## 2 Structural Considerations

**Figure 1:**
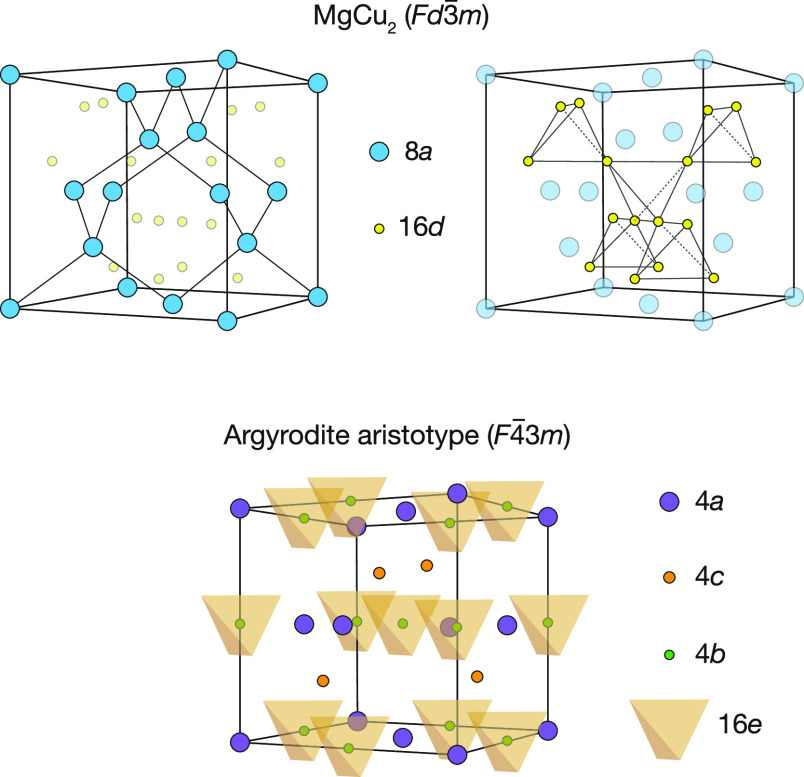
Top panel: the MgCu_2_ structure with Mg (blue) occupying 8a sites and Cu (yellow) occupying 16d sites. The center of each 16d tetrahedra is a vacant 8b site. Bottom panel: the cubic argyrodite aristotype. Half of the *Fd*3̅*m* 8b sites are occupied by P, becoming 4b sites in the reduced-symmetry *F*4̅3*m* space group, while the *Fd*3̅*m* 8a sites are split into symmetry inequivalent *F*4̅3*m* 4a and 4c sites.^35,36^

The Li_6_PS_5_*X* argyrodites typically adopt a cation-disordered cubic aristotype in the *F*4̅3*m* space group, which can be considered to be derived from the MgCu_2_ cubic Laves phase (*Fd*3̅*m* space group).^21,35,37^ In MgCu_2_, the Mg sites (8a) form a diamond-structured array, and the Cu sites (16d) form an interpenetrating corner-sharing network of tetrahedra (Figure 1, upper panel). In Li_6_PS_5_X, the phosphorus atoms occupy only half of the “Cu” tetrahedra, reducing the crystal symmetry from *Fd*3̅*m* to *F*4̅3*m*. The “Cu” sites (now denoted 16e) are fully occupied by S, forming a face-centered cubic array of PS_4_ tetrahedra, and the “Mg” sites (now split into 4a and 4c) are occupied by an equal ratio of S and X anions (Figure 1, lower panel).^35,36^ In Li_6_PS_5_I, the anions are ordered, with I atoms fully occupying the 4a sites and S atoms fully occupying the 4c sites. In Li_6_PS_5_Cl and Li_6_PS_6_Br, the Cl/Br and S atoms are disordered, with both anions distributed over the 4a and the 4c sites.

**Figure 2:**
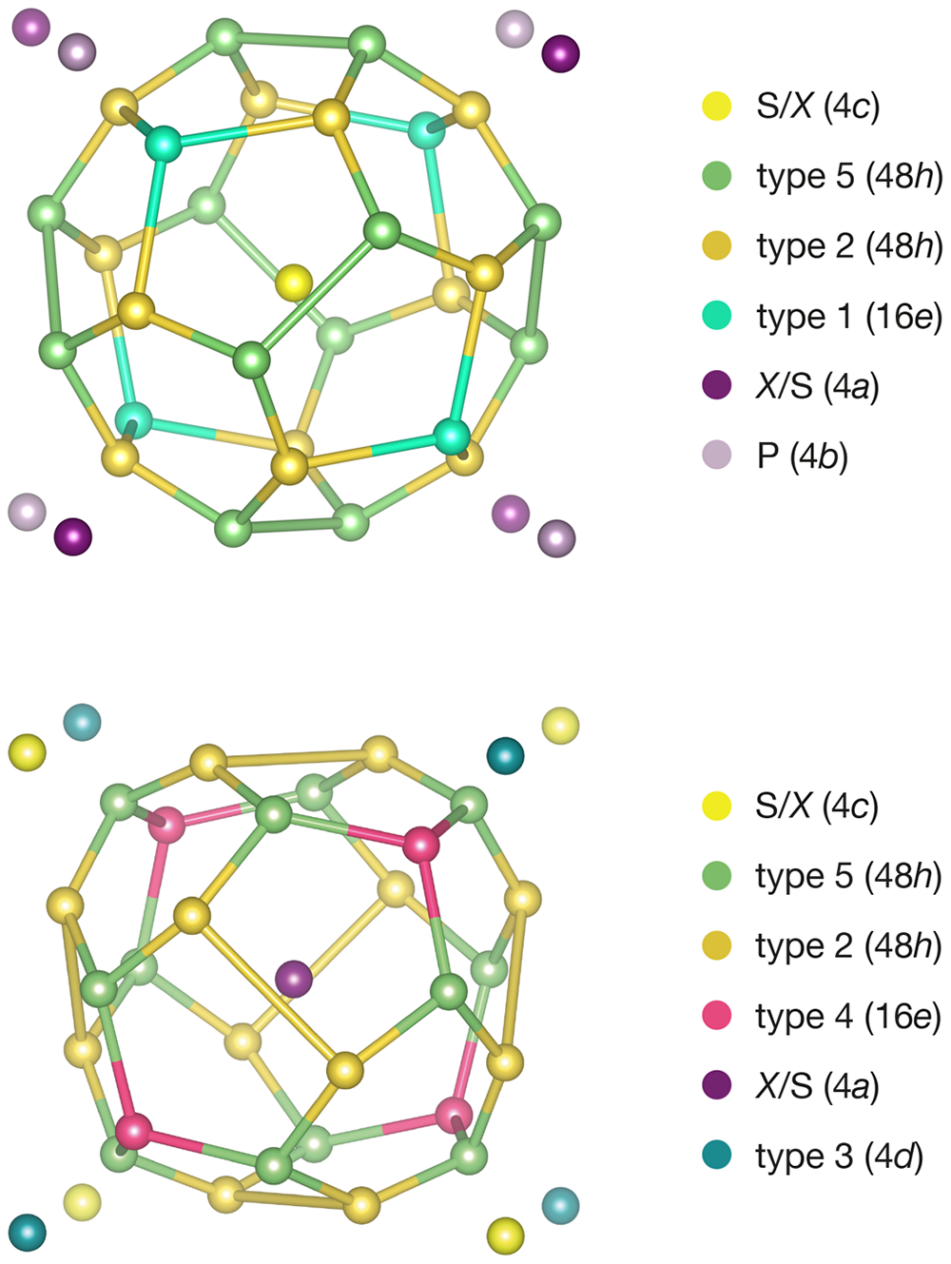
(Top) The centers of the 28 tetrahedral sites surrounding each 4c position. These define a truncated triakis tetrahedron,^40^ centered inside a cube of neighboring 4a and 4c sites. (Bottom) Each 4a site is coordinated by a topologically identical set of sites, centered inside a cube of 4a and 4b sites. The lithium-site coordination polyhedra around neighboring 4a and 4c sites are linked by shared hexagonal faces consisting of type 2 and type 5 positions.

This three-dimensional arrangement of anions at 4a, 4c, and 16e positions defines a tetrahedrally close-packed lattice.^37−39^ The centers of these tetrahedra represent interstitial sites available to accommodate immobile cations (such as P) or mobile lithium ions. In MgCu_2_, the Mg and Cu positions define the vertices of three crystallographically distinct tetrahedral sites. The lower crystal symmetry of the argyrodites splits these into six distinct tetrahedral types, which are listed in Table 1, and were first described by Deiseroth et al.^36^ In Li_6_PS_5_X, one set of tetrahedra (type 0) is occupied by phosphorus, while the remaining tetrahedra (types 1–5) are available to potentially accommodate lithium. The type 3 tetrahedra are centered on the 4d Wyckoff positions with four 16e sites as vertices, which they share with the type 0 PS_4_ tetrahedra. The remaining tetrahedra types 1, 2, 4, and 5 form face-sharing cages around the 4a and 4c S/X sites. These cages each contain 28 tetrahedra, and each tetrahedron represents one lithium interstitial site (Figure 2). The 4a and 4c coordination polyhedra are topologically identical: each has 12 pentagonal faces and four hexagonal faces, with the face-centers forming a 16-vertex Frank–Kasper polyhedron.^35,40^ The hexagonal faces of these coordination polyhedra are arranged tetrahedrally around each central S/X site and are comprised of alternating type 2 and type 5 tetrahedral sites, which are shared between adjacent 4a and 4c-coordination polyhedra.

**Table 1:** Tetrahedral Holes Formed by the Close-Packed S/X Anion Substructure in Li_6_PS_5_X (*F*4̅3*m*, Setting 2), Following the Classification of Deiseroth et al.^36^

type	Wyckoff notation for tetrahedron center	Comments
0	4b (P)	Center of PS_4_ tetrahedra
1	16e	4-fold coordination of 4c sites.
2	48h	12-fold coordination of 4a and 4c sites. Form face-sharing pairs around 4a sites.
3	4d	Four common corners with neighboring PS_4_ tetrahedra.
4	16e	4-fold coordination of 4a sites.
5	48h	12-fold coordination of 4c and 4a sites. Form face-sharing pairs around 4c sites.

In high-temperature modifications of Li_6_PS_5_X, lithium is disordered over the available tetrahedral sites types 1–5. X-ray single-crystal data for high-temperature-Li_6_PS_5_I show that electron density associated with these disordered lithium ions is smeared out over an extended region, but is predominantly associated with type 5 tetrahedra.^21,36^ Subsequent neutron diffraction studies have typically assigned Li in Li_6_PS_5_X as primarily occupying either 48h sites—located within the type 5 tetrahedra—or 24g sites—located at the shared face between adjacent type 5 tetrahedral pairs,^23,27,41^ and denoted as type 5a by Deiseroth et al.^36^ The standard model for lithium diffusion in Li_6_PS_5_X considers only these type 5 48h and type 5a 24g positions, with microscopic lithium motion assumed to occur as a sequence of stochastic “jumps” between these sites.^23,28,30,32,42−44^ Because type 5 tetrahedra form disconnected face-sharing pairs, a description of lithium transport that only considers the type 5 and type 5a sites are necessarily incomplete: any lithium motion beyond simple hopping back-and-forth within paired type 5 sites must involve other tetrahedral site types.^45^

The capacity for nontype 5 tetrahedra to accommodate lithium may, therefore, determine the degree to which lithium can diffuse through the structure. Some computational evidence for the role of nontype 5 tetrahedra in lithium diffusion in Li_6_PS_5_X argyrodites comes from previous bond-valence calculations, which predict three distinct lithium sites.^26,27,29^ Nontype 5 sites have also been identified in recent neutron diffraction studies of Li_6_PS_5_Br and Li_6_PS_5_Cl,^46,47^ as well as in lithium-argyrodites with lithium stoichiometries *x*(Li) > 6.^48−50^ A general mechanistic description of lithium conduction in lithium-argyrodites that describes the role of different lithium sites and that can explain the relationship between substitutional anion disorder and fast lithium transport, however, is currently lacking.

## 3 Methods

To simulate lithium dynamics in Li_6_PS_5_I and Li_6_PS_5_Cl, we have performed a series of ab initio molecular dynamics simulations using the Vienna ab initio simulation package.^51,52^ For all calculations, we have used the revised Perdew–Burke–Ernzerhof generalized gradient approximation PBEsol exchange–correlation functional.^53^ Interactions between core and valence electrons were described using the projector augmented wave (PAW) method,^54^ with cores of [He] for Li, [Ne] for P, [Ne] for S, [Ne] for Cl, and [Kr] for I. Zero-pressure volumes were calculated for ordered Li_6_PS_5_I and Li_6_PS_5_Cl, with the 4c sites occupied by S, and the 4*a* sites occupied by I or Cl. These calculations consisted of full geometry optimizations for a single unit cell (52 atoms) starting from the Materials Project structure ID-985592,^55^ with a cutoff of 700 eV, and a 2 × 2 × 2 Monkhorst–Pack *k*-point mesh. The optimized lattice parameters were then used to construct 2 × 2 × 2 supercells (416 atoms) for the subsequent molecular dynamics simulations.

The molecular dynamics simulations used a plane-wave cutoff of 280 eV and only the gamma point for *k*-space sampling. All MD simulations were performed at 500 K, and used a time-step of 2 fs. For both Li_6_PS_5_I and Li_6_PS_5_Cl, we have considered three different S/X configurations: 0% site-inversion, with S fully occupying the 4c sites and X fully occupying the 4a sites, corresponding to the experimentally reported ordered Li_6_PS_5_I structure; 50% site-inversion, with a random S/X configuration that approximates the experimentally reported disordered Li_6_PS_5_Cl structure; and 100% site-inversion, with S fully occupying the 4a sites and X fully occupying the 4c sites. The same randomly generated 50% site-inverted S/X configuration was used for the Li_6_PS_5_I 50% and Li_6_PS_5_Cl 50% simulations. For each system, the lattice parameters were kept fixed to the zero-pressure 0% optimized values. For each MD simulation, two equilibration stages were performed, first using a 2 ps NVE run with temperature rescaling every 50 steps, followed by a 2 ps *NVT* run. For each simulation, the production runs were 70 ps.

The analysis of MD simulation trajectories is often complicated by “trivial” thermal motions of the mobile ions and of the host framework. Here, we are interested in nontrivial lithium displacements that contribute to net lithium diffusion, rather than short-timescale vibrational motion. To help resolve the lithium-diffusion processes in our simulations, we have extracted a series of “inherent” structures^56−58^ from each simulation trajectory by performing conjugate-gradient geometry optimizations for configurations selected every 50 time-steps. Each inherent structure represents a local minimum on the corresponding 3N-dimensional potential energy surface, and the sequences of inherent structures from a given simulation describe the nontrivial motion of lithium ions as they move between these local minima.

A dataset containing inputs and outputs for all DFT calculations supporting this study is available under the CC BY-SA 4.0 license from the University of Bath Research Data Archive.^59^ All codes used to analyze the simulation trajectories and to generate the corresponding figures are available as a series of Jupyter notebooks^60^ under the MIT license. Our analysis used the Matplotlib,^61^ NumPy,^62^ Pymatgen,^63,64^ SciPy,^65^ tqdm,^66^ vasppy,^67^ site-analysis,^68^ polyhedral-analysis,^69^ Kinisi,^70^ and crystal-torture^71^ Python packages.

## 4 Results

### 4.1 Lithium Mean-Squared Displacements

**Figure 3:**
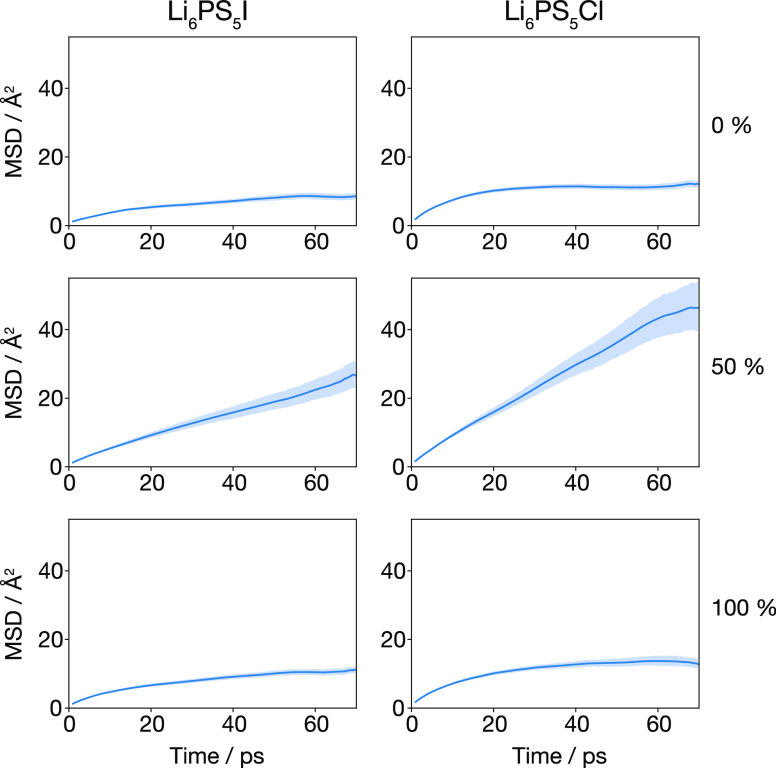
MSD of lithium ions for Li_6_PS_5_I and Li_6_PS_5_Cl with 0%, 50%, and 100% S/X site inversion. Shaded regions show estimated 95% confidence intervals, calculated at each time interval via bootstrap sampling.^73^ Figure adapted from ref (60) under a CC BY-SA 4.0 license https://creativecommons.org/licenses/by-sa/4.0.

The rate at which individual lithium ions diffuse through a solid electrolyte is described by the lithium self-diffusion coefficient, which can be calculated from molecular dynamics simulations as the slope of the lithium mean-squared displacement (MSD) versus time, in the long time limit.^72^Figure 3 shows calculated lithium MSD for Li_6_PS_5_I and Li_6_PS_5_Cl with 0, 50, and 100% S/X site inversion. For both X = I and X = Cl, for the anion-ordered systems (0 and 100% site inversion), the MSD initially increases, before plateauing at longer times, giving an effective lithium diffusion coefficient of zero. These plateaus indicate that in the anion-ordered systems the lithium ions do not diffuse freely, but instead are confined to small disconnected regions of space. The MSDs of the anion-disordered systems (50% site inversion) show qualitatively different behavior: these MSDs continually increase at long times, corresponding to nonzero diffusion coefficients and long-ranged lithium diffusion. These results are consistent with data from previous molecular dynamics simulations,^22,30−32^ and highlight two interesting points. First, the diffusion behavior is qualitatively the same for X = I and X = Cl, as noted previously by Stamminger et al.^30^ Second, lithium caging is observed for both 0 and 100% site inversion, showing that long-ranged lithium diffusion is not a first-order consequence of occupying 4a sites with sulfur, but that instead anion disorder across the 4a and 4c sites is the necessary prerequisite.

### 4.2 Tetrahedral Site Occupations

The qualitative difference in diffusion behavior between anion-ordered and anion-disordered Li_6_PS_5_X suggests that the arrangement of anions in each system directs the microscopic lithium diffusion mechanism. To examine the relationship between the anion configuration and diffusion behavior, we can calculate the time-averaged tetrahedral site-type populations for each simulation trajectory. To assign lithium ions to specific sites at each time-step, we use the instantaneous positions of the S/X anions to define the tetrahedra vertices. A lithium ion is deemed to occupy a particular tetrahedron, if it sits inside the volume defined by these vertex positions.^74^

**Figure 4:**
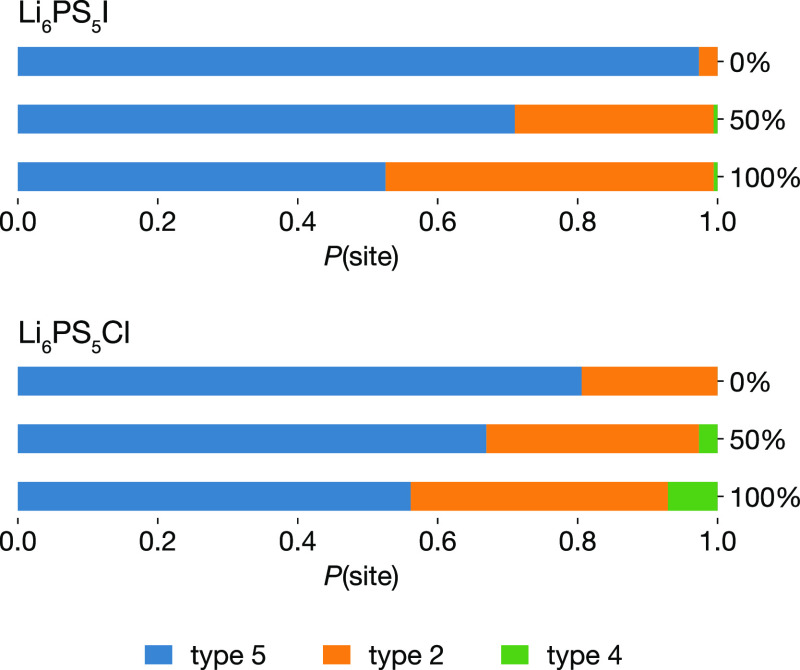
Time-averaged probabilities for a lithium ion to occupy a particular tetrahedral site type, for (a) Li_6_PS_5_I and (b) Li_6_PS_5_Cl with 0%, 50%, and 100% S/X site inversion. Figure adapted from ref (60) under a CC BY-SA 4.0 license https://creativecommons.org/licenses/by-sa/4.0.

Figure 4 shows the time-averaged probabilities for a lithium ion to occupy each of the six tetrahedral site types, calculated using the inherent structures from each simulation trajectory. Each atomic configuration used in this analysis, therefore, corresponds to a local potential energy minimum. For all systems, lithium ions are most likely to occupy type 5 tetrahedra. This is broadly consistent with previous diffraction studies of Li_6_PS_5_I (anion-ordered) and Li_6_PS_5_Cl (anion-disordered), which have assigned lithium as predominantly occupying two positions associated with the type 5 tetrahedra: the 48h positions located inside each type 5 tetrahedron, and the 24g positions (type 5a sites) in the trigonal faces shared by type 5 tetrahedra pairs.^21,36,75^

In all six systems, we find some proportion of lithium ions located at nontype 5 tetrahedra. For 0% site-inverted Li_6_PS_5_I, lithium partially occupies tetrahedra types 5 and 2. In all the other systems, lithium is distributed over tetrahedra types 5, 2, and 4. The possibility of lithium occupying nontype 5 tetrahedra in Li_6_PS_5_X argyrodites has been discussed in detail by Deiseroth et al.,^36^ who noted that lithium ions must pass through nontype 5 tetrahedra for long-ranged lithium transport to occur.^45^

The observation of partial occupation of nontype 5 tetrahedra is qualitatively consistent with recent neutron diffraction studies of Li_6_PS_5_Br and Li_6_PS_5_Cl, which have reported partial occupation of type 2 tetrahedra.^46,47^ The study of Minafra et al. also reported data for Li_6_PS_5_I,^46^ with lithium assigned only to type 5 and type 5a sites, in apparent contradiction to the simulation results presented here. Experimental samples of Li_6_PS_5_I are fully anion-ordered, and are approximated by our 0% site inversion model. For this system, our simulations predict only 2.5% of Li occupies type 2 sites, which is unlikely to be resolved in diffraction experiments. Similarly, we predict very low numbers of lithium ions occupy type 4 sites, making direct experimental observation challenging.

In each system, the partially occupied tetrahedra define the set of pathways available for possible lithium diffusion. The relationship between the degree of anion-site inversion and lithium diffusion (cf. Figure 3), however, is not explained by the varying occupations of these tetrahedral sites (Figure 4). All six models predict partial occupation of both type 5 and type 2 tetrahedra, with type 2 occupation increasing with greater anion site-inversion. The set of all type 5 and type 2 tetrahedra forms a three-dimensional network of face-sharing tetrahedra, and we might, therefore, expect all systems to exhibit long-ranged lithium diffusion. Yet, this is not the case, as only the 50% site-inverted systems exhibit long-ranged lithium diffusion. All systems apart from 0% site-inverted Li_6_PS_5_I also exhibit partial type 4 tetrahedral occupation, which further increases the connectivity of the three-dimensional tetrahedral network (Figure 6a), and provides additional potential pathways for long-ranged diffusion. As is the case for increasing the occupation of type 2 tetrahedra, an increase in the occupation of the type 4 occupation is similarly not correlated with increased lithium diffusion. The 100% site-disordered Li_6_PS_5_X models have the highest probabilities of occupying both type 2 and type 4 tetrahedra, yet exhibit no long-ranged Li diffusion.

**Figure 5:**
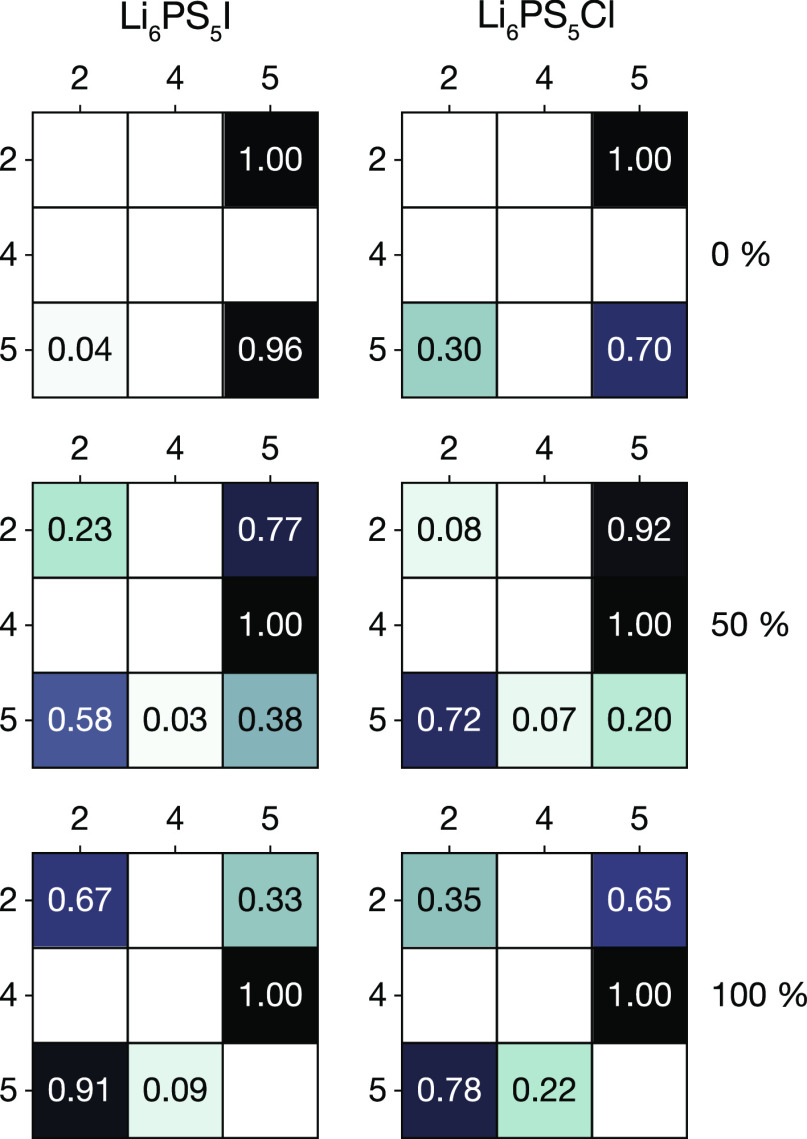
Transition matrices showing the probabilities for lithium ions to move from site type *i* (rows) to site type *j* (columns), in 0%, 50%, and 100% site-inverted Li_6_PS_5_X. Each row sums to a total probability of 1.0. Individual probabilities are shown rounded to two decimal places. Figure adapted from ref (60) under a CC BY-SA 4.0 license https://creativecommons.org/licenses/by-sa/4.0.

**Figure 6:**
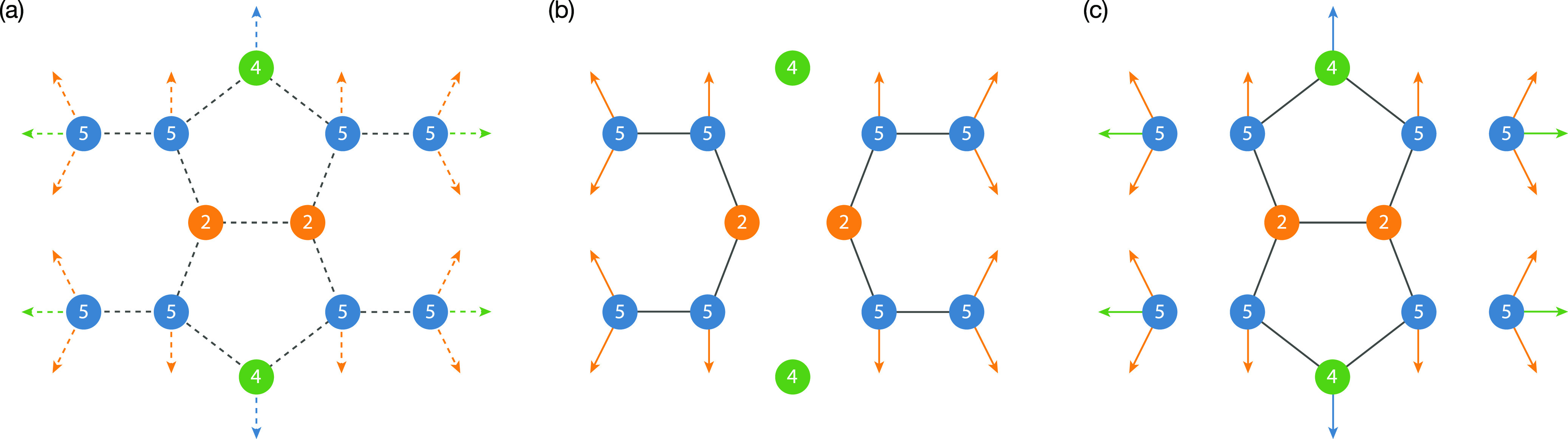
(a) Schematic of all possible site–site lithium diffusion pathways between face-sharing pairs of type 2, type 4, and type 5 tetrahedra. (b) In 0% site-inverted Li_6_PS_5_I and Li_6_PS_5_Cl, only 5 → 5, 5 → 2, and 2 → 5 transitions are observed, corresponding to “caged” diffusion around 4c sites. (c) In 100% site-inverted Li_6_PS_5_I and Li_6_PS_5_Cl, only 2 → 2, 2 → 5, 5 → 2, 5 → 4, and 4 → 5 transitions are observed, corresponding to “caged” diffusion around 4a sites.

### 4.3 Site–Site Transition Probabilities

The lack of direct correlations between the tetrahedral site-type occupations and the calculated diffusion data indicates that the varying capacities for long-ranged diffusion in the Li_6_PS_5_X argyrodites are not a simple consequence of whether lithium does or does not partially occupy nontype 5 sites. Instead, we consider the possibility that it is not simply the occupations of the different tetrahedral sites that is important, but that the anion configuration may crucially affect whether the diffusion pathways connecting these sites are active or inactive. To determine the active diffusion paths in each system, we have analyzed our inherent structure trajectories to identify transition events, defined as a lithium ion moving from one tetrahedral site to a neighboring site. We can then calculate the probability that a lithium ion initially occupying site type *i* subsequently moves to another site of type *j*, averaged over all observed transitions, for each *i* → *j* pairing. Figure 5 shows transition matrices of the probabilities *P*(*i* → *j*). In each matrix, each row corresponds to a different initial site type (2, 4, or 5) and each nonblank entry in that row gives the observed probability of moving to a given adjacent site type. For 0% anion site-inversion, only 5 → 5, 5 → 2, and 2 → 5 transitions occur. With no 2 → 2 or 5 → 4 transitions, no long-ranged diffusion is possible, and lithium motion is restricted to closed “cages” around the 4c sites (see Figure 6b).^76^ For 100% site-inversion, we observe only 2 → 2, 5 → 2, and 5 → 4 transitions. Long-ranged diffusion is now blocked by the inactive 5 → 5 transition, again leading to restricted lithium diffusion around the 4a sites (see Figure 6c). For 50% site-inversion, however, all jump types are observed, which is consistent with the existence of a contiguous diffusion network that can accommodate long-ranged Li diffusion; Li can now move around 4a sites and 4c sites. We, therefore, find that lithium motion between different tetrahedral sites is dependent on the local S/X anion configuration, which gives rise to a qualitative difference in active lithium diffusion pathways between anion-ordered and anion-disordered Li_6_PS_5_X systems, as well as between models with 0 and 100% site inversion.

### 4.4 Time-Average Site Positions and Site–Site Percolation

**Figure 7:**
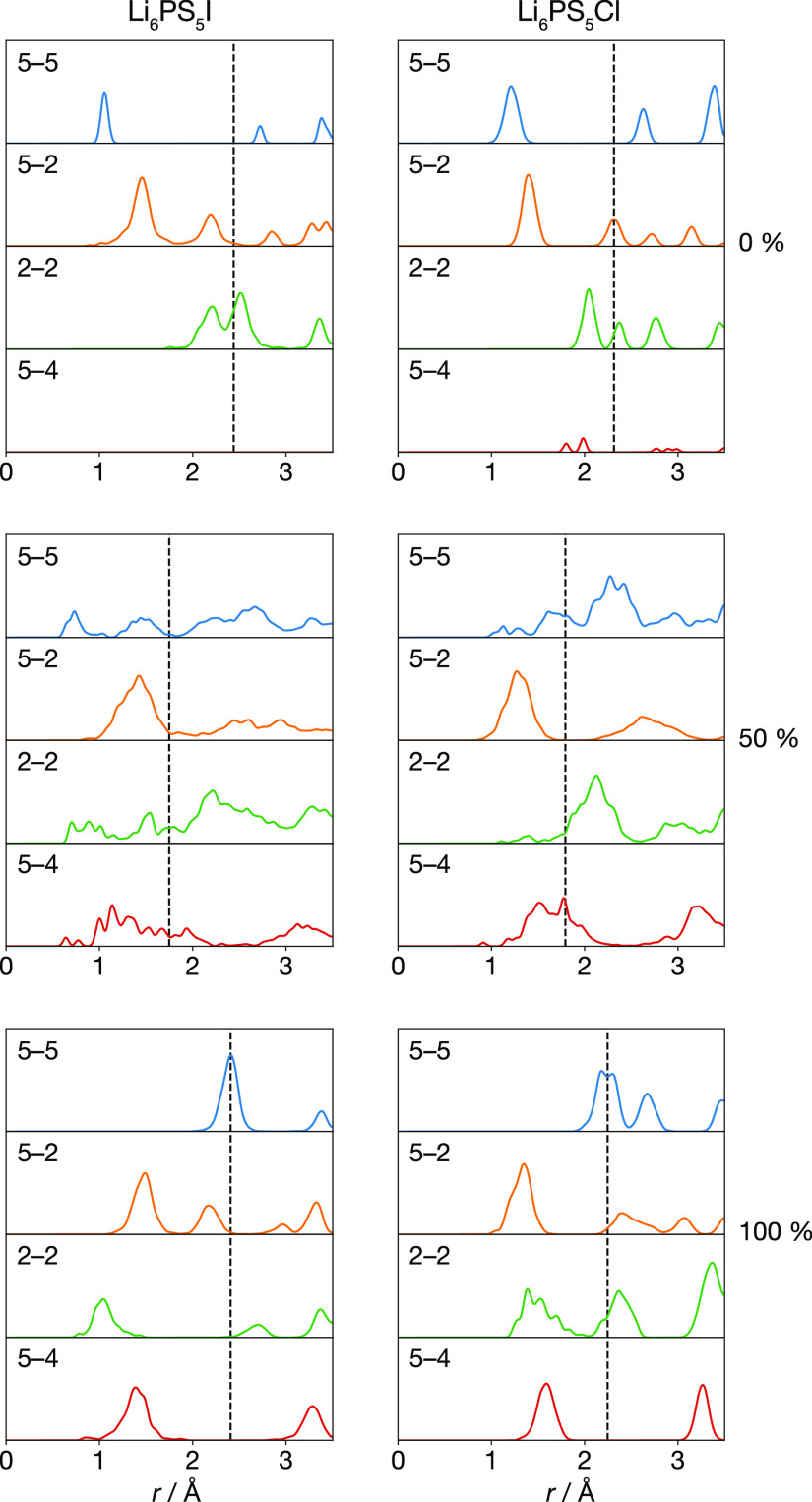
Lithium site–site radial distribution functions for Li_6_PS_5_I and Li_6_PS_5_Cl at 0%, 50%, and 100% anion-site-inversion. For each set of data, the vertical dashed line shows the minimum separation for which the lithium sites form a percolating network. Figure adapted from ref (60) under a CC BY-SA 4.0 license https://creativecommons.org/licenses/by-sa/4.0.

As discussed in Section 2, all argyrodites possess topologically identical MgCu_2_-structured anions, and therefore have equivalent tetrahedral interstitial sites available for lithium diffusion. Understanding why a specific arrangement of anions across the 4a and 4c sites gives continuous versus discontinuous diffusion pathways requires going beyond the analysis presented above, which only considers the occupation of specific tetrahedra and the movement of lithium between these discrete sites. In the mixed-anion Li_6_PS_5_X argyrodites, each tetrahedral hole may have a mixture of S and X anions at its vertices, giving an asymmetric coordination environment. The equilibrium lithium position within a given tetrahedron therefore may not be located at the “ideal” tetrahedron center. To understand how the S/X configuration affects the lithium substructure, we have calculated average lithium positions within each tetrahedron from our intrinsic structure trajectories. We have then considered the distributions of site–site distances for each Li_6_PS_5_X simulation. The radial distribution functions, *g*(*r*), for specific pairs of these per-site average lithium positions, are shown in Figure 7.

For the 0% site-inverted systems, the site–site rdfs show sharp peaks, indicating a degree of ordering by the lithium ions, and a clear hierarchy of site separations: the shortest 5–5 separation is ∼1.2 Å, corresponding to pairs of adjacent type 5 tetrahedra. The next-nearest separation is 5–2 at ∼1.5 Å, and the first 2–2 site separation peak is at >2 Å. The 100% site inverted systems also show sharp rdf peaks and distinct short and long site–site separations. Now, the nearest-neighbor distances increase in the order 2–2 < 5–4 < 5–2 < 5–5, with the first 5–5 peak at > 2 Å. Comparing the positions of the nearest-neighbor first peak for each site-pair to the corresponding site–site transition probabilities above (Figure 5) shows long intersite distances correspond to “inactive” diffusion paths, while short intersite distances correspond to “active” diffusion paths. The site–site rdfs for the 50% site-inverted systems show broader distributions, indicating a range of site–site separations and a somewhat disordered lithium substructure. We find minimum intersite distances of <1 Å for all four site–site distances. This does not mean that all site–site distances are this short in the 50% disordered system. Instead, we observe a continuous range of short to long separations, indicating that these anion-disordered systems the average lithium positions within each site are statically disordered.

In each system, the hierarchy of site–site distances (Figure 7) is correlated with the pattern of active and inactive lithium–site-transitions described in the previous section (Figure 5). In the anion-ordered 0 and 100% site-inverted systems, tetrahedral pairs with short site–site distances exhibit active transitions, while transitions between tetrahedral pair types with longer nearest-neighbor distances are inactive. In the anion-disordered 50% site-inverted systems, all combinations of face-sharing tetrahedral pairs exhibit a range of short and long site–site distances, and all site–site transitions are observed.

This correlation between short or long site–site distances and active or inactive lithium-site-transitions suggests a model wherein fast long-ranged lithium diffusion in the anion-disordered systems is associated with a percolating network of short lithium site separations, while nondiffusive motion in the anion-ordered systems is associated with a nonpercolating disconnected network. To test this model, for each system we have calculated the minimum site–site separation distance at which the average lithium positions at each site form a percolating network (Figure 7). We find this threshold percolation distance is significantly shorter for the anion-disordered systems than for the anion-ordered systems. For both the 0% and 100% site-inverted systems, the large 2–2 or 5–5 separations mean lithium motion is predominantly constrained to local “cages” of closely separated sites, surrounding the 4c or 4a positions, respectively.

### 4.5 Anion–Lithium Radial Distribution Functions

**Figure 8:**
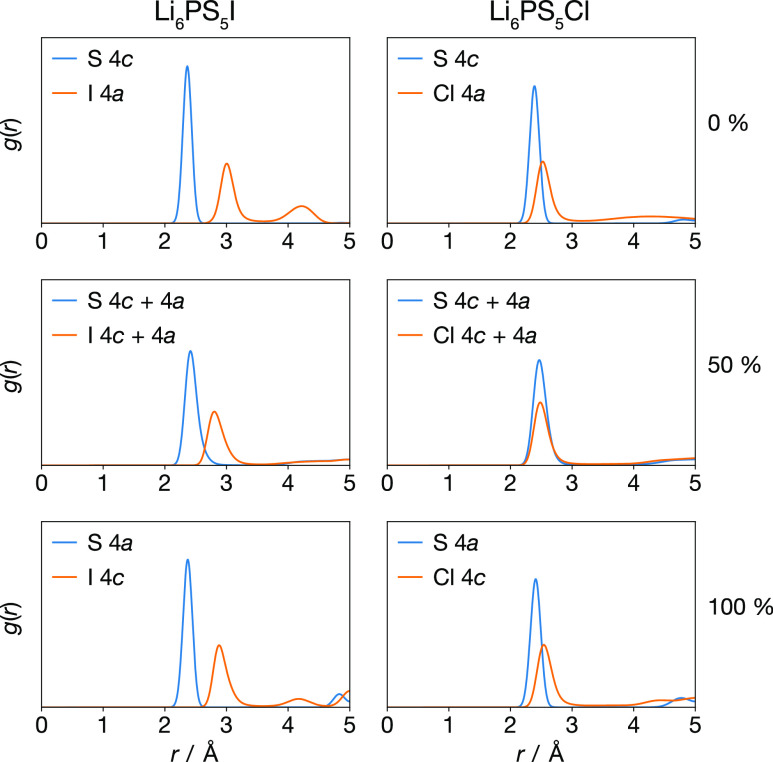
S(4a/4c)–Li and X(4a/4c)–Li radial distribution functions, *g*(*r*), for Li_6_PS_5_I and Li_6_PS_5_Cl with 0%, 50%, and 100% S/X site inversion. Figure adapted from ref (60) under a CC BY-SA 4.0 license https://creativecommons.org/licenses/by-sa/4.0.

The effect of ordered versus disordered anion configurations on the lithium substructure is also evident in the S(4c/4a)–Li and X(4a/4c)–Li radial distribution functions (Figure 8). In the anion-ordered 0% and 100% site-inverted systems, the nearest-neighbor S–Li distances are shorter than the nearest-neighbor X–Li distances. This corresponds to a displacement of the Li site positions toward the S-occupied 4c or 4a sites, and can be understood in terms of simple electrostatics—positive lithium ions are more strongly attracted to S^2–^ ions than to X^–^ ions. This asymmetry in anion–lithium coordination is larger for X = I than for X = Cl because of the additional difference in anion ionic radii. Because the S and X anions are crystallographically ordered in these systems, a decrease of Li-site distances to S-occupied 4c or 4a sites corresponds to an increase of Li-site distances to X-occupied 4a or 4c sites, respectively. This pattern of short S–Li and long X–Li distances, combined with the S/X ordering over 4c and 4a sites, explains the Li–Li site distances discussed in the previous section (Figure 7), which then explains the pattern of active and inactive lithium diffusion pathways in these systems (Figure 6). This effect, where an ordered S/X anion substructure induces ordered displacements in the lithium-site positions, is illustrated schematically in Figure 10a,b.

**Figure 9:**
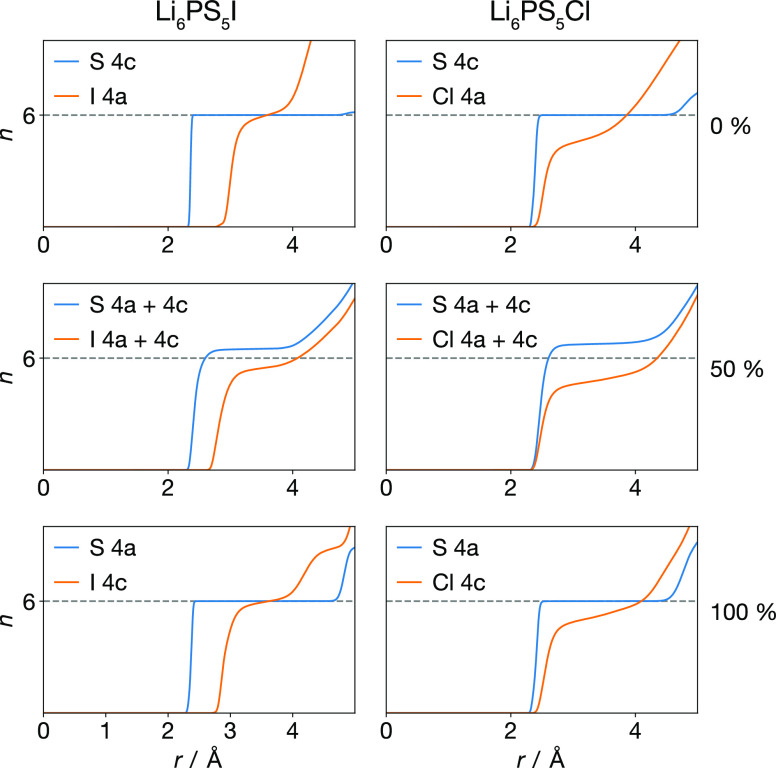
S(4a/4c)–Li and X(4a/4c)–Li coordination numbers, *n*, for Li_6_PS_5_I and Li_6_PS_5_Cl with 0%, 50%, and 100% S/X site inversion. Figure adapted from ref (60) under a CC BY-SA 4.0 license https://creativecommons.org/licenses/by-sa/4.0.

**Figure 10:**
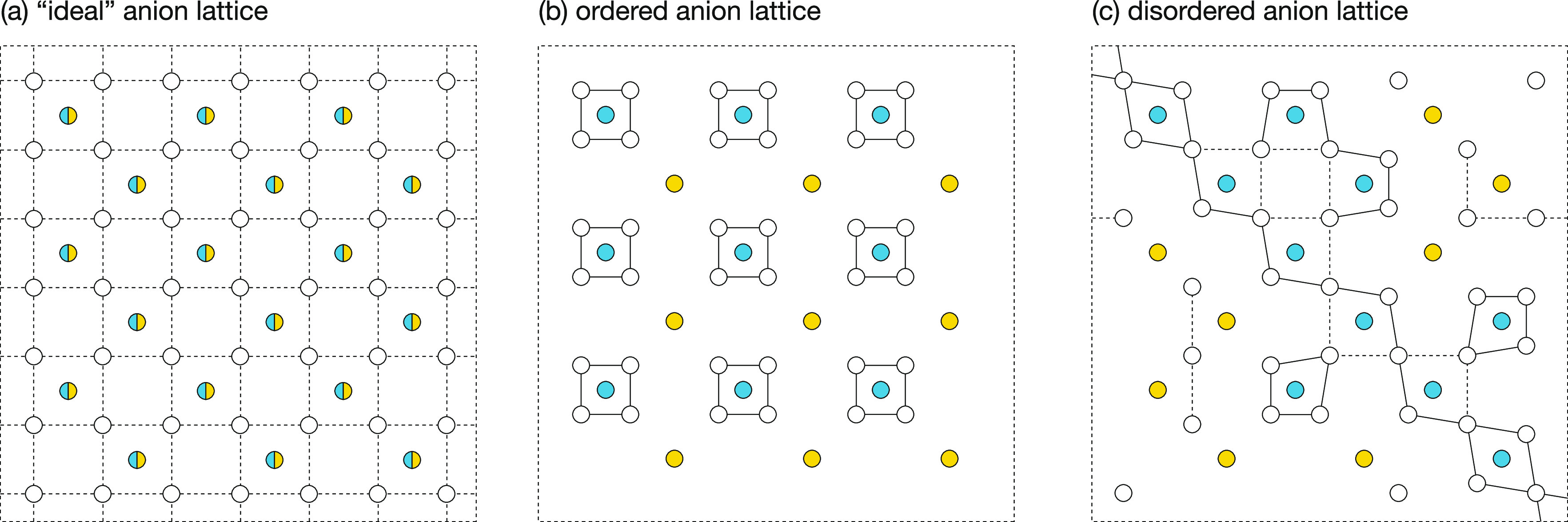
Schematic illustrating the coupling between configurational order/disorder of the anion substructure and static order/disorder of the Li sites. (a) “Ideal” anion substructure. All Li sites are in symmetric environments, and all site–site distances are equal. (b) Ordered anion substructure. All Li sites are in locally asymmetric environments, and move toward S^2–^ (blue) and away from X^–^ (yellow) anions, to give an ordered nonpercolating network of short site–site distances. (c) Disordered anion substructure. The shift of Li site positions depends on the local anion configuration. Moving toward S and away from X anions produces a disordered percolating network of short site–site distances. Solid lines in (c) indicate site–site distances that are shorter than in the “ideal” anion substructure.

In the anion-disordered 50% site-inverted systems, the S–Li and X–Li nearest-neighbor distances are more similar, and the corresponding peaks are broader. In particular, for 50% site-inverted Li_6_PS_5_Cl, the S–Li and X–Li nearest-neighbor peaks coincide. In an anion-disordered system, the Li sites experience a range of local coordination environments with different permutations of S and X neighboring anions. The average position of each Li site now depends on the specific local anion environment. Because the S and X anions are disordered, the arrangement of long Li site–site distances, which correspond to inactive diffusion paths, is also disordered, allowing a percolating network of shorter active diffusion paths (Figure 10c).

The average numbers of lithium ions around the S/X 4a and 4c sites can be calculated by integrating the rdf data (Figure 9). For Li_6_PS_5_X stoichiometry argyrodites there are exactly six lithium ions per 4a/4c S anion. For all the anion-ordered systems (0% and 100% site-inversion), we find an average of *n* = 6 lithium ions around the 4c or 4a S atoms, respectively, suggesting the structure can be described as 4c or 4a-centered SLi_6_ subunits, with X occupying the remaining 4a or 4c sites. For the 50% systems, we find an average of *n* > 6 Li ions associated with each S atom, suggesting a more complex lithium arrangement. Because the ratio of lithium to 4a/4c sulfur is consistently 6 Li to 1 S in all cases, an average coordination number of *n* > 6 Li ions indicates that some Li contributes to coordination of more than one S center.

### 4.6 Sulfur–Lithium Coordination Polyhedra

For a more detailed description of the local S–Li coordination environments, we have classified the local lithium coordination around each 4a or 4c sulfur according to the degree of geometric similarity with respect to a set of reference SLi_*x*_ coordination polyhedra. We consider a “coordination polyhedron” to consist of a single S atom residing at a 4c or 4a site plus the set of lithium ions within a spherical cutoff *r*_coord_, with the cutoff distance chosen to lie in the first plateau region from Figure 9. For each coordination polyhedron, we can quantify the geometric similarity to a given reference polyhedron, such as a perfect octahedron, by calculating the corresponding CSM.^77^ The CSM can be thought of as a normalized “distance” between two polyhedral geometries: larger CSM values indicate larger deviations from the reference geometry. Here, we classify each coordination polyhedron geometry by computing CSM values with respect to a set of common polyhedral coordination motifs^64,78^ and selecting the “most similar” motif—given by the smallest CSM.

**Figure 11:**
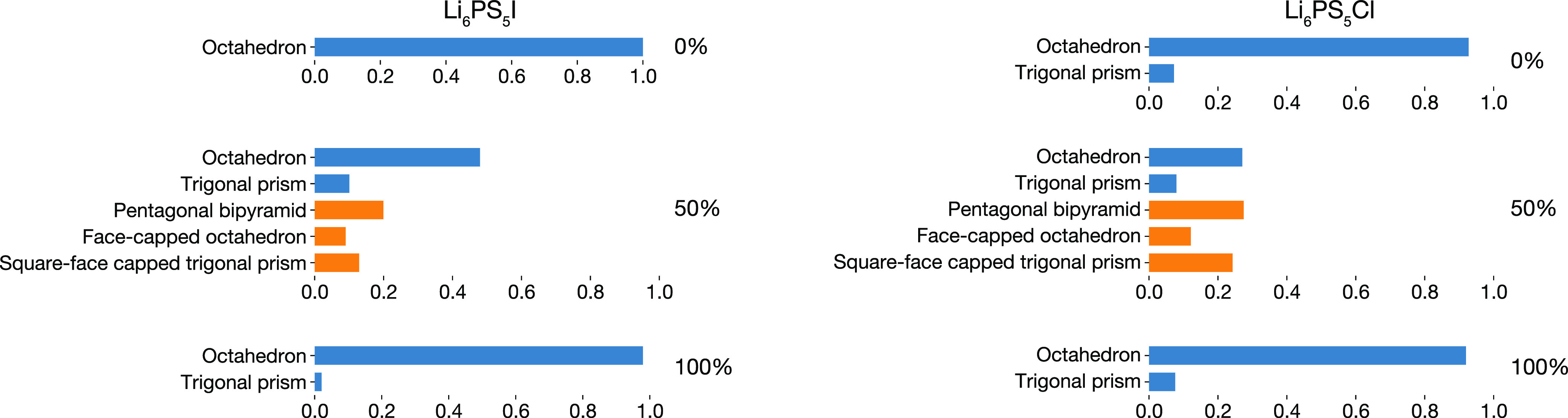
Populations of SLi_*x*_ coordination polyhedral geometries, for Li_6_PS_5_I and Li_6_PS_5_Cl with 0%, 50%, and 100% S/X site inversion. Polyhedral geometries have been assigned by calculating the minimum continuous symmetry measure (CSM)^77^ for a set of reference coordination geometries.^64,78^ Blue bars denote 6-coordinate polyhedra; yellow bars denote 7-coordinate polyhedra. Figure adapted from ref (60) under a CC BY-SA 4.0 license https://creativecommons.org/licenses/by-sa/4.0.

The relative proportions of different coordination polyhedral geometries are shown in Figure 11. For the 0% and 100% site-inverted systems, we observe nearly exclusively 6-coordinate polyhedra,^79^ as suggested by the average S–Li coordination numbers (cf. Figure 9). In these anion-ordered systems, these SLi_6_ units preferentially adopt approximately octahedral geometries, with a few trigonal prismatic configurations observed for all but the Li_6_PS_5_I 0% system. An octahedral distribution of lithium ions around each S is reasonable from electrostatics, because it minimizes the net Coulomb repulsion between the lithium ions for *n* = 6. For the anion-disordered systems, we observe a mixture of 6- and 7-coordinate polyhedra, in agreement with the average *n* > 6 coordination number obtained from the *g*(*r*) data.

### 4.7 SLi_*x*_ Polyhedra Dynamics

The SLi_*x*_ coordination polyhedra provide a schema for classifying the lithium dynamics in each system. For each polyhedron, we consider two features: the first is the set of lithium ions that define the polyhedron vertices, and the second is the set of edges that connect these vertices, which then defines the polyhedron topology. In our simulation trajectories, each lithium ion is assigned an integer index. The set of lithium ions that define a specific coordination polyhedron (all those within *r*_coord_ of the central atom) can be described by a vertex list of these ion indices, for example, (1, 3, 7, 20, 52, and 100). The edge topology connecting these ions is described by an undirected edge graph, where we consider an edge formed between any two vertices of a polyhedron with a separation smaller than a threshold distance *r*_edge_. These features allow us to define three classes of lithium motion:

1. Neither the vertex list nor the edge graph change, but the polyhedron undergoes a “rigid” rotation in space.
2. Only the edge graph changes. The vertex list remains unchanged. This corresponds to some internal reorganization of lithium ions that changes the polyhedron topology.
3. The vertex list changes (and the edge graph therefore also changes). This corresponds to a lithium ion leaving a polyhedron (moving beyond the cutoff *r*_coord_), or joining a new polyhedron, or both.

The first two of these correspond to local lithium motion, while the third constitutes lithium transfer between SLi_*x*_ polyhedra, which is required for long-ranged lithium diffusion.

In the anion-ordered 0% and 100% site-inverted systems, we find that exchange of lithium ions between coordination polyhedra is nearly never observed on the timescale of our simulation,^80^ which is consistent with the long-time plateaus in the lithium MSD data and the inactive site–site transitions described above, and with data from previous molecular dynamics simulations of anion-ordered Li_6_PS_5_X argyrodites.^22,30−33^ In these anion-ordered systems, the lithium dynamics nearly exclusively comprises internal motions of SLi_6_ units.

**Figure 12:**
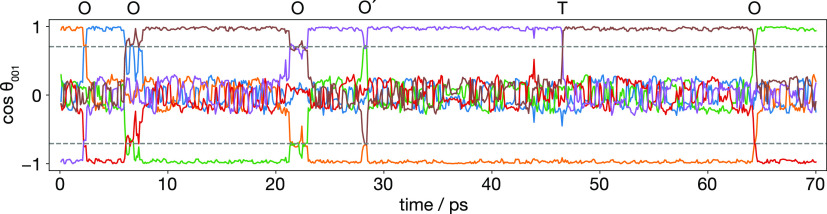
Internal dynamics of one 4c SLi_6_ coordination polyhedron in the Li_6_PS_5_I 0% site-inverted system at 500 K. The ordinate gives the normalized projection of each S–Li vector onto the [001] cell axis (i.e., the cosine similarity). The horizontal dashed lines show cos(±45°). Labels above the plot indicate different classes of internal dynamics: O indicates a “rigid” octahedral rotation (Figure 13a); O′ is an “incomplete” rotation, that moves to a 45° rotated orientation, before returning to the previous geometry; T indicates an internal reorganization via a trigonal-prismatic intermediate (Figure 13b). Both the octahedral rotations and trigonal-prismatic rearrangements have stable intermediates where multiple lithium ions occupy type 2 tetrahedra. Figure adapted from ref (60) under a CC BY-SA 4.0 license https://creativecommons.org/licenses/by-sa/4.0.

**Figure 13:**
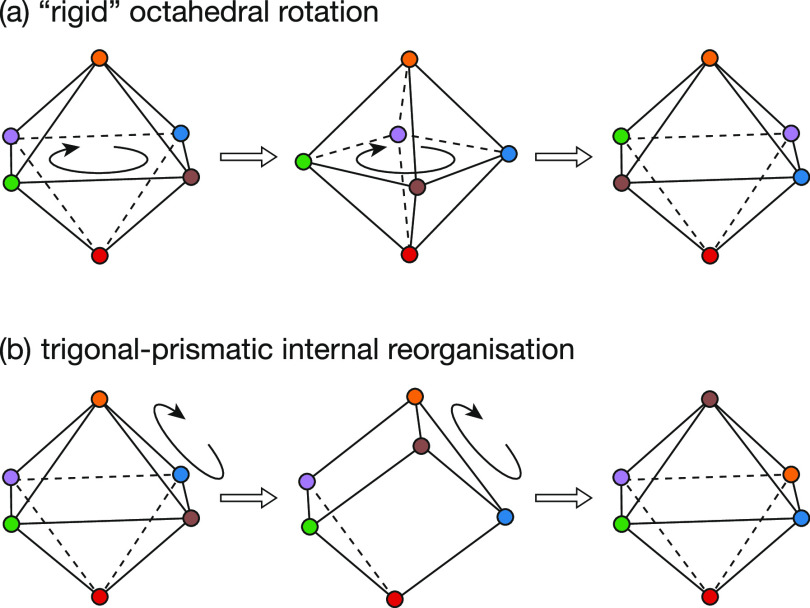
Schematic of the concerted (a) rigid rotation and (b) trigonal-prismatic reorganization modes of lithium motion within SLi_6_ coordination polyhedra in the anion-ordered Li_6_PS_5_X systems.

For anion-ordered Li_6_PS_5_I (Figure 12), these motions are predominantly rigid rotations of the SLi_6_ octahedral coordination polyhedra, which proceed via a concerted motion of four coplanar lithium ions around the perpendicular axis (Figure 13). We also observe a small number of internal reorganizations consisting of octahedral → trigonal-prismatic → octahedral transitions, which proceed via the concerted motion of three face-sharing lithium ions (Figure 13b). This internal reorganization via a trigonal-prismatic intermediate is analogous to a “Bailar twist” and is the *minimum distortion pathway* between two topologically inequivalent octahedra.^81^ Both these motions have midpoints that are local potential energy minima, where the displaced lithium ions have moved from type 5 to type 2 tetrahedral sites.

For anion-ordered Li_6_PS_5_Cl we observe similar behavior, with local Li dynamics comprising both rigid octahedral rotations and internal reorganization via trigonal-prismatic intermediates. These local Li dynamics in Li_6_PS_5_Cl are more frequent than in Li_6_PS_5_I, and individual SLi_*x*_ units spend more time in intermediate configurations, where some lithium ions occupy type 2 sites, making it difficult to classify discrete dynamical events.

For the anion-disordered Li_6_PS_5_I and Li_6_PS_5_Cl systems, we find qualitatively different behavior to the anion-ordered systems described above. With S/X disorder present, lithium ions undergo rapid exchange between SLi_*x*_ units. This, again, is consistent with the MSD and site–site transition analyses presented above, and with previous molecular dynamics simulations.^22,30−33^

### 4.8 String-Like Collective Diffusion

The conventional model for ionic diffusion in solid electrolytes assumes that ion transport is effected by a sequence of single-ion “hops” between discrete sites,^82−84^ and this model has been assumed in the analysis of diffusion in lithium argyrodites in a number of previous studies.^9,11,22−25,28,41−44,48,85^ For many fast-ion solid electrolytes, however, ion transport instead proceeds via collective diffusion processes, whereby multiple ions participate in synchronous cooperative motion.^86−96^ Such cooperative motions can be considered a defining characteristic of “superionic” conductivity, in distinction to fast, but conventional, single-particle-hopping,^88^ and concerted lithium diffusion has recently been proposed to be a contributing factor in the exceptionally high ionic conductivities of Li-excess Li_6+*x*_M_*x*_Sb_1–*x*_S_5_I (M = Si, Sn, Ge) argyrodites.^48^

Our site–site transition analysis, above, allowed a characterization of the connectivity of active diffusion pathways within the argyrodite structure, and how the topology of the diffusion network varies with the 4a/4c S/X anion configuration. That analysis, however, cannot distinguish between a single-particle hopping mechanism, and a collective “superionic” diffusion mechanism. In the anion-ordered system, we have already observed that the internal dynamics of SLi_6_ coordination polyhedra is highly concerted—octahedral rotations and trigonal-prismatic reorganizations both involve synchronous motion of groups of lithium ions. For the anion-disordered systems, however, the more complex lithium dynamics means that this local “lithium-coordination” analysis is less useful in distinguishing between individual or concerted lithium motion.

To determine whether the lithium ions in anion-disordered Li_6_PS_5_X systems diffuse via individual or collective processes, we have identified groups of lithium ions that are involved in cooperative string-like motions.^97^ We define strings that form on a timescale Δ*t* by connecting two mobile ions *i* and *j* if
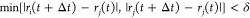
1

This corresponds to selecting pairs of mobile ions where one ion has moved into a position previously occupied by the second ion. To construct strings, we then connect ion pairs that occur within the same time window that contain one common mobile ion.

The identification of strings of mobile ions, via eq 1, is not on its own sufficient to distinguish between individual hopping and a concerted diffusion mechanism. In the case of simple vacancy hopping, a sequence of “vacancy hops” produces a string of mobile ions, even though in this case the single-ion hops that produce this sequence are temporally uncorrelated. To distinguish a temporally uncorrelated process from a correlated process, we consider the distribution of string lengths observed in time Δ*t*. For a stochastic hopping process, the number of hops in time window Δ*t*, and hence the distribution of string lengths, samples a Poisson distribution.^98^ Observing a probability distribution of string lengths that strongly deviates from a Poisson distribution is, therefore, evidence for ion displacements that are clustered in time.

**Figure 14:**
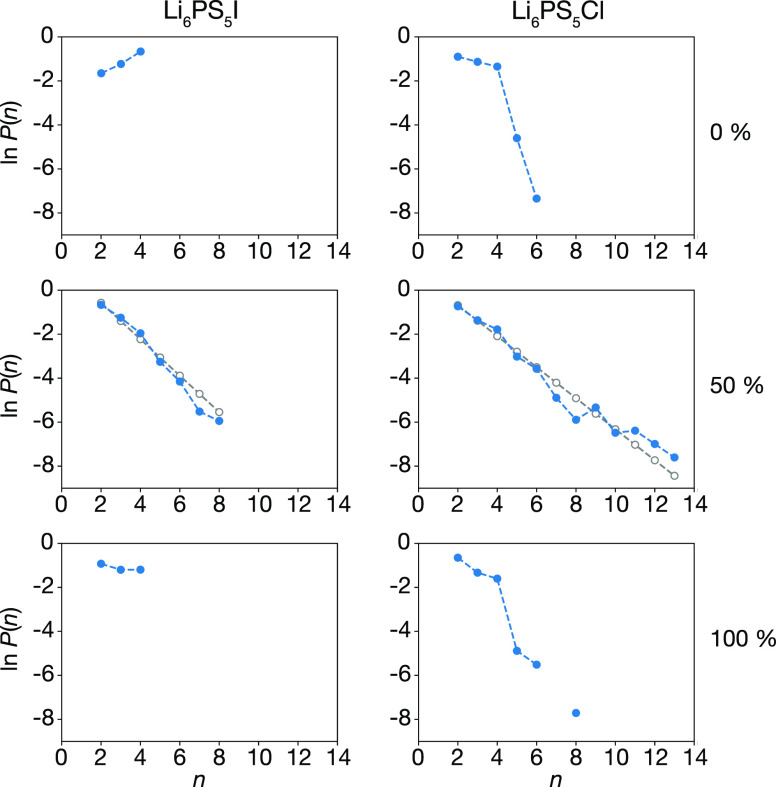
Probability distributions *P*(*n*) of string lengths *n* for Li_6_PS_5_I and Li_6_PS_5_Cl with 0%, 50%, and 100% S/X site inversion. For the 50% site-inverted systems, the gray empty symbols show the maximum likelihood estimate for a geometric distribution for each dataset. Figure adapted from ref (60) under a CC BY-SA 4.0 license https://creativecommons.org/licenses/by-sa/4.0.

Figure 14 shows the probability distributions of string lengths, *P*(*n*), from our simulations, for Δ*t* = 5 ps. For the anion-ordered systems, we find high probabilities of strings with lengths 2–4, corresponding to the concerted motions of ions within SLi_6_ coordination polyhedra described above. For the 50% site-inverted systems, we observe a range of string lengths, with *P*(*n*) following an approximate geometric distribution. This mirrors the behavior observed in supercooled glassy liquids^97^ where string-like diffusion is often associated with *dynamic heterogeneity*,^56,99−101^ whereby spatially correlated subsets of particles exhibit much faster dynamics than the system average. A geometric distribution of string lengths is consistent with a mechanistic model consisting of string “initiation”, followed by string “propagation” with the probability of a string increasing in length from *N* particles to *N* + 1 particles is independent of *N*. Our analysis here indicates that lithium mobility is effected by concerted ion motions in all our systems. In the anion-ordered systems, these motions are “closed-loops” typically consisting of groups of four or three lithium ions undergoing local cyclic motions (cf. Figure 13). In the anion-disordered systems, we find string-like concerted motions that facilitate the diffusion of extended groups of lithium ions.

## 5 Summary and Discussion

The data from our molecular dynamics simulations provide multiple complementary perspectives of the atomic-scale diffusion behavior in Li_6_PS_5_X argyrodites. Combining these perspectives provides the basis of a coherent model for the lithium diffusion mechanism in these systems, and how this is affected with the degree of substitutional 4a/4c anion disorder.

We first analyzed the lithium distribution in each simulation in terms of occupation of the different tetrahedral sites within the close-packed anion substructure. We find that lithium ions predominantly occupy type 5 tetrahedral sites; particularly so for the fully ordered S(4c)/X(4a) systems; which broadly agrees with previous diffraction experiments.^21,36^ For all systems, we also find partial occupation of nontype 5 tetrahedra. Deiseroth et al. have previously noted that long-ranged diffusion of lithium through the argyrodite structure requires movement of lithium through non type-5 tetrahedra.^45^ In more recent studies of lithium argyrodites, however, the lithium substructure has typically been considered purely in terms of occupation of type 5 (and 5a) sites, with lithium diffusion decomposed into classes of direct 5 → 5 transitions.^9,11,22,24,25,27,30,32,33,41,42,85,102,103^ This simplified perspective neglects the different tetrahedra types defined by the close-packed anion substructure, and obscures their roles in the lithium diffusion processes in different argyrodite compositions. For Li_6_PS_5_X systems, these nontype 5 tetrahedra are not simply high-energy intermediates that define local potential energy maxima along lithium diffusion 5 → 5 pathways. Instead, we find stable Li configurations, corresponding to local potential-energy minima, in which nontype 5 sites are occupied. This illustrates the importance of these sites when describing the relevant potential energy surface for lithium diffusion. Occupation of nontype 5 tetrahedra has been identified in experimental samples for *x*(Li) > 6 argyrodites^48−50^ as well as in recent neutron diffraction studies of Li_6_PS_5_Br and Li_6_PS_5_Cl.^46,47^ The tetrahedrally close-packed geometry defined by the MgCu_2_-structured anion sites is common to all argyrodites, and we, therefore, expect analyses that consider diffusion in terms of ion motion between these different tetrahedra to provide useful insight into the transport mechanisms operating in argyrodite stoichiometries beyond Li_6_PS_5_X.

In the case of the Li_6_PS_5_X systems, an analysis in terms of occupation of spatially discrete lithium sites also allows the active lithium diffusion pathways in each system to be resolved. While the partial site-occupations for these anion-ordered and anion-disordered systems might equally suggest the existence of a contiguous lithium-diffusion pathway, via a network of face-sharing tetrahedra, lithium does not always move freely between these partially occupied tetrahedra. For anion-ordered systems, there are regular “blocked” pathways between specific neighboring tetrahedral sites, giving a noncontiguous diffusion network. Our analysis of the time-average lithium positions within each partially occupied tetrahedral site shows that “blocked” pathways correspond to large average Li–Li separations, while the set of short “active” pathways forms closed orbits around the 4c or 4a S anions, producing the restricted “cage-like” lithium diffusion reported in previous studies.^22,30,31,34^ This can be considered a form of lithium ordering, induced by the configurational anion order, that arises from a preference for shorter S–Li than X–Li distances. In the anion-disordered systems, conversely, we find no systematic pattern of short–long average lithium separations between adjacent sites: the set of short “active” pathways is disordered and forms a percolating three-dimensional network, that facilitates long-ranged lithium diffusion.

The idea that the lithium ions are, in some sense, ordered when the S/X anions are substitutionally ordered, but that lithium ions are disordered when these anions are disordered, and that this difference directs the lithium diffusion behavior, also emerges from our analysis of the local lithium coordination environment around the 4a and 4c S anions. In the anion-ordered systems, the lithium substructure can be considered as 6-coordinate SLi_*x*_ polyhedra around the 4c or 4a sulfur atoms. This “pseudo-ordering” corresponds to an effective crystal symmetry where the regular 6-coordinate SLi_*x*_ motif is invariant under integer lattice-vector translations, even though the lithium ions are crystallographically disordered over the available tetrahedral sites.

**Figure 15:**
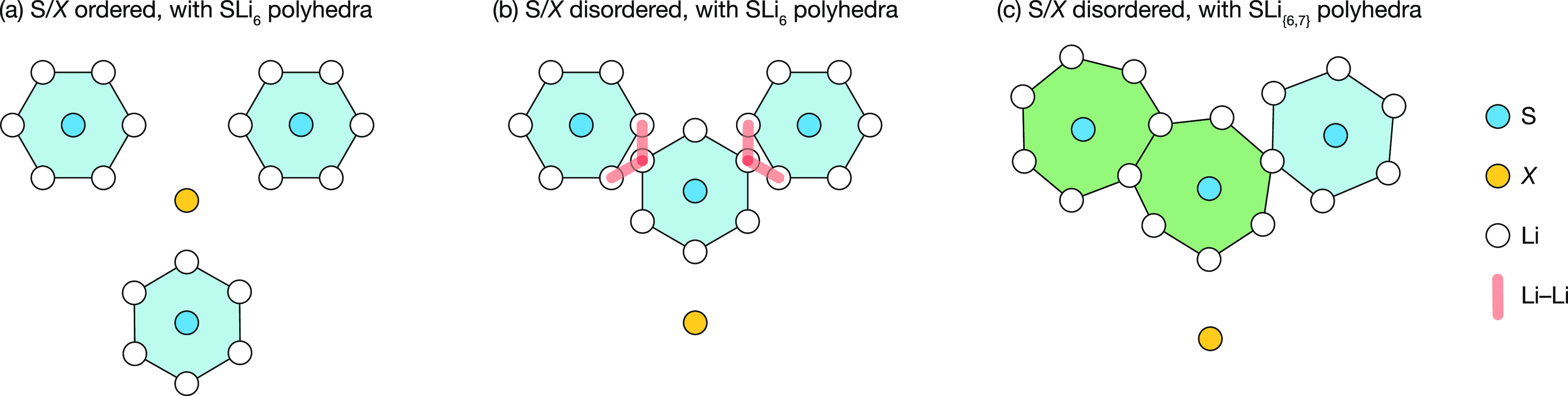
Schematic of the change in lithium coordination around 4a/4c S ions as a function of S/X order/disorder. (a) For S/X anion ordering, the 4c (or 4a) S ions are well separated, and Li ions arrange into SLi_*x*_ coordination “cages”. (b) S/X anion disorder means S ions occupy adjacent 4a and 4c sites. Maintaining SLi_6_ coordination would produce short Li–Li distances (red arrows), which are Coulombically disfavored. (c) Sharing Li between adjacent 4a and 4c S coordination environments reduces the net Li–Li repulsion, and gives a mix of irregular SLi_6_ and SLi_7_ coordination environments.

The pseudo-ordering of lithium in S/X-ordered argyrodites can be explained by considering the various Coulombic interactions between S–Li, X–Li, and Li–Li ion pairs. Lithium ions are attracted more strongly to S^2–^ than X^–^ anions, because of the larger formal charge of S, which encourages Li to adopt cage-like configurations around the 4a or 4c S ions. The repulsive Li–Li Coulombic interactions, however, tend to maximize Li–Li separations within each coordination cage. In an anion-ordered system, these two factors can be simultaneously optimized by arranging exactly six Li ions around each 4a or 4c S anion (Figure 15a). Within each SLi_6_ unit, the Li–Li repulsion is minimized by the Li ions adopting an approximately octahedral configuration. Lithium motions that only produce internal reorganization of individual SLi_6_ units do not disrupt this pseudo-ordering, and are frequent on a simulation timescale. These motions are highly cooperative, proceeding via octahedral or trigonal-prismatic intermediates that preserve the mutual Li–Li separation.

Long-ranged diffusion, in contrast, requires lithium motion between adjacent SLi_*x*_ coordination polyhedra. Consider two adjacent [SLi_6_ + SLi_6_] polyhedra in the ordered system. Lithium transfer between these polyhedra produces a [SLi_5_ + SLi_7_] configuration, and disrupts the preferred pseudo-ordered coordination motif. This “disordered” configuration is energetically disfavored, because of the increased Li–Li repulsion within the SLi_7_ unit. Forming SLi_*x*_ coordination environments with *x* ≠ 6 in these anion-ordered Li_6_PS_5_X argyrodites can, therefore, be considered a form of defect-pair formation, analogous to Frenkel pair formation in stoichiometric crystals.^98^

In the S/X-disordered systems, we can again understand the behavior by considering the interplay of anion–Li and Li–Li Coulomb interactions. Li ions again preferentially coordinate S, rather than X anions. We have seen in the anion-ordered system that if these SLi_*x*_ units are well-separated, the Li–Li interactions between SLi_*x*_ units are negligible, and the total electrostatic energy is minimized by forming a set of regular octahedral SLi_6_ subunits. In an anion-disordered system, however, S ions occupy both 4a and 4c sites. Forming adjacent SLi_6_ octahedra would now introduce short Li–Li distances between Li ions nominally associated with the different S anions (Figure 15b): SLi_6_ configurations can be said to be “geometrically frustrated”.^104^ To avoid these short Li–Li separations, Li ions can instead be “shared” between adjacent SLi_*x*_ environments, producing a mixture of SLi_6_ and SLi_7_ coordination motifs, and spatially disordered average Li positions within each tetrahedral site (Figure 15c). Lithium motion between different S-coordination environments is now possible without changing the net distribution of coordination environments—for example, Li transfer between two adjacent SLi_*x*_ environments might proceed as SLi_7_ + SLi_6_ → SLi_6_ + SLi_7_. This is expected to correspond to a low-energy diffusion process, analogous to diffusion of extrinsic defects (vacancies or interstitials) in a nonstoichiometric conventional crystal.^98^

Superionic conductivity in solid electrolytes is often associated with some form of disorder within the mobile-ion substructure,^105−108^ and studies of various solid electrolyte families have revealed a range of mechanisms that can contribute to this substructural disorder.^10,32,104,109−113^ The results presented here provide another example of this general principle. In this instance, superionic conductivity arises as a consequence of substitutional disorder within the immobile ion host-substructure, via induced mobile-ion disorder. Enhanced conductivities have been reported in other materials with configurational host-framework disorder,^114−118^ and the same underlying mechanism of induced mobile-ion disorder may be responsible in these cases. This raises the question of the extent to which substitutional framework-disorder might be useful as a general design strategy to obtain superionic conductivity in other families of solid electrolytes?

In the case of the lithium argyrodites, we can ask the more specific question of how these results for Li_6_PS_5_X might suggest design strategies for optimizing the ionic conductivity of *x*(Li) ≠ 6 argyrodites? Our results indicate that in the case of Li_6_PS_5_X, substitutional anion disorder, and the resulting lithium disorder, are key to achieving fast Li diffusion. This suggests that substitutional disorder; either due to mixed anions, or to partial substitution of P with elements such as Ge, might have a similar positive effect in *x*(Li) ≠ 6 systems. A disordered potential energy surface may promote fast diffusion by causing concerted “superionic” diffusion mechanisms,^119^ rather than slower independent-hopping diffusion mechanisms. A second, contrasting, interpretation comes from noting that in Li_6_PS_5_X systems, S/X anion disorder gives S occupying adjacent 4a and 4c sites, which contributes to lithium disorder because of Coulombic frustration of otherwise regular SLi_*x*_ units. This perspective suggests that in *x*(Li) ≠ 6 argyrodites, fast lithium-ion diffusion might be achieved in compositions in which the S/X ratio differs from one, making, for example, Li_7_PS_6_ an interesting end-member case.

Finally, we note that the highest room-temperature ionic conductivities for lithium argyrodites have been reported for “Li excess” systems with *x*(Li) > 6, such as Li_6.6_P_0.4_Ge_0.6_S_5_I^11^ and Li_6+*x*_M_*x*_Sb_1–*x*_S_5_I (M = {Si,Sn,Ge}).^48^ In the latter case, lithium has been shown to occupy nontype 5 sites, which was attributed to the *x*(Li) > 6 lithium stoichiometry, with this lithium “site-disorder” suggested as the origin of the observed fast lithium-ion conduction.^48^ This suggestion is consistent with the general principle that lithium disorder (in some form) is necessary to achieve fast lithium diffusion, and raises the possibility of doing so directly through control of lithium stoichiometry; in contrast to the induced lithium disorder arising from substitutional framework disorder, as for the anion-disordered Li_6_PS_5_*X* systems. Attributing the exceptional ionic conductivity of these experimental *x*(Li) > 6 systems to a single mechanistic origin is challenging. The excess Li stoichiometry is a consequence of aliovalent substitution of host-framework atoms occupying the 4b site, which introduces a new source of substitutional disorder within the host framework. These materials also exhibit small amounts of S/I disorder, which may also contribute to Li disorder and enhance Li diffusion.^11^ Resolving the interplay between stoichiometry, structure, and lithium dynamics, and using this understanding to further optimize the ionic conductivities of this family of solid electrolytes presents an intriguing challenge. The complexity of these issues suggests that a full understanding will only be reached by combining data from systematic experimental studies of controlled stoichiometries with insight from corresponding computational studies.

## Supporting Information Available

Comparison of unprocessed simulation trajectories versus inherent structure trajectories for site analysis (PDF)

## Notes

A dataset containing inputs and outputs for all DFT calculations supporting this study is available under the CC BY 4.0 license from the University of Bath Research Data Archive.^59^ All codes used to analyze the simulation trajectories and to generate the corresponding figures are available as a series of Jupyter notebooks as ref (60) under the MIT license.

## References

[ref1] Zhang Z.; Shao Y.; Lotsch B.; Hu Y.-S.; Li H.; Janek J.; Nazar L. F.; Nan C.-W.; Maier J.; Armand M.; Chen L. New horizons for inorganic solid state ion conductors. Energy Environ. Sci. 2018, 11, 1945 10.1039/c8ee01053f.

[ref2] Bachman J. C.; Muy S.; Grimaud A.; Chang H.-H.; Pour N.; Lux S. F.; Paschos O.; Maglia F.; Lupart S.; Lamp P.; Giordano L.; Shao-Horn Y. Inorganic solid-state electrolytes for lithium batteries: Mechanisms and properties governing ion conduction. Chem. Rev. 2016, 116, 140 10.1021/acs.chemrev.5b00563. 26713396

[ref3] Ohno S.; Banik A.; Dewald G. F.; Kraft M. A.; Krauskopf T.; Minafra N.; Till P.; Weiss M.; Zeier W. G. Materials design of ionic conductors for solid state batteries. Prog. Energy 2020, 2, 022001 10.1088/2516-1083/ab73dd.

[ref4] Famprikis T.; Canepa P.; Dawson J. A.; Islam M. S.; Masquelier C. Fundamentals of inorganic solid-state electrolytes for batteries. Nat. Mater. 2019, 18, 1278. 31427742 10.1038/s41563-019-0431-3

[ref5] Janek J.; Zeier W. G. A solid future for battery development. Nat. Energy 2016, 1, 16141 10.1038/nenergy.2016.141.

[ref6] Culver S. P.; Koerver R.; Krauskopf T.; Zeier W. G. Designing ionic conductors: The interplay between structural phenomena and interfaces in thiophosphate-based solid-state batteries. Chem. Mater. 2018, 30, 4179 10.1021/acs.chemmater.8b01293.

[ref7] Squires A. G.; Scanlon D. O.; Morgan B. J. Native defects and their doping response in the lithium solid electrolyte Li_7_La_3_Zr_2_O_12_. Chem. Mater. 2019, 32, 1876 10.1021/acs.chemmater.9b04319.

[ref8] Fuchs T.; Culver S. P.; Till P.; Zeier W. G. Defect-mediated conductivity enhancements in Na_3–*x*_Pn_1–*x*_W_*x*_S_4_ (Pn = P, Sb) using aliovalent substitutions. ACS Energy Lett. 2020, 5, 146 10.1021/acsenergylett.9b02537.

[ref9] Schlem R.; Ghidiu M.; Culver S. P.; Hansen A.-L.; Zeier W. G. Changing the static and dynamic lattice effects for the improvement of the ionic transport properties within the argyrodite Li_6_PS_5–*x*_Se_*x*_I. ACS Appl. Energy Mater. 2019, 3, 9 10.1021/acsaem.9b01794.

[ref10] Zhou L.; Assoud A.; Shyamsunder A.; Huq A.; Zhang Q.; Hartmann P.; Kulisch J.; Nazar L. F. An entropically stabilized fast-ion conductor: Li_3.25_[Si_0.25_P_0.75_]S_4_. Chem. Mater. 2019, 31, 7801 10.1021/acs.chemmater.9b00657.

[ref11] Kraft M. A.; Ohno S.; Zinkevich T.; Koerver R.; Culver S. P.; Fuchs T.; Senyshyn A.; Indris S.; Morgan B. J.; Zeier W. G. Inducing high ionic conductivity in the lithium superionic argyrodites Li_6+*x*_P_1–*x*_Ge_*x*_S_5_I for all-solid-state batteries. J. Am. Chem. Soc. 2018, 140, 16330 10.1021/jacs.8b10282. 30380843

[ref12] Zhang Y.; He X.; Chen Z.; Bai Q.; Nolan A. M.; Roberts C. A.; Banerjee D.; Matsunaga T.; Mo Y.; Ling C. Unsupervised discovery of solid-state lithium ion conductors. Nat. Commun. 2019, 10, 5260 10.1038/s41467-019-13214-1. 31748523 PMC6868160

[ref13] Kahle L.; Marcolongo A.; Marzari N. High-throughput computational screening for solid-state Li-ion conductors. Energy Environ. Sci. 2020, 13, 928 10.1039/c9ee02457c.

[ref14] Muy S.; Voss J.; Schlem R.; Koerver R.; Sedlmaier S. J.; Maglia F.; Lamp P.; Zeier W. G.; Shao-Horn Y. High-throughput screening of solid-state Li-ion conductors using lattice-dynamics descriptors. iScience 2019, 16, 270 10.1016/j.isci.2019.05.036. 31203184 PMC6581664

[ref15] Sendek A. D.; Cubuk E. D.; Antoniuk E. R.; Cheon G.; Cui Y.; Reed E. J. Machine learning-assisted discovery of solid Li-ion conducting materials. Chem. Mater. 2018, 31, 342 10.1021/acs.chemmater.8b03272.

[ref16] Krauskopf T.; Culver S. P.; Zeier W. G. Bottleneck of diffusion and inductive effects in Li_10_Ge_1–*x*_Sn_*x*_P_2_S_12_. Chem. Mater. 2018, 30, 1791 10.1021/acs.chemmater.8b00266.

[ref17] Weiss M.; Weber D. A.; Senyshyn A.; Janek J.; Zeier W. G. Correlating transport and structural properties in Li_1+*x*_Al_*x*_Ge_2–*x*_(PO_4_)_3_ LAGP prepared from aqueous solution. ACS Appl. Mater. Interfaces 2018, 10, 10935 10.1021/acsami.8b00842. 29516733

[ref18] Krauskopf T.; Muy S.; Culver S. P.; Ohno S.; Delaire O.; Shao-Horn Y.; Zeier W. G. Comparing the descriptors for investigating the influence of lattice dynamics on ionic transport using the superionic conductor Na_3_PS_4–*x*_Se_*x*_. J. Am. Chem. Soc. 2018, 140, 14464 10.1021/jacs.8b09340. 30284822

[ref19] Burbano M.; Carlier D.; Boucher F.; Morgan B. J.; Salanne M. Sparse cyclic excitations explain the low ionic conductivity of stoichiometric Li_7_La_3_Zr_2_O_12_. Phys. Rev. Lett. 2016, 116, 135901 10.1103/physrevlett.116.135901. 27081991

[ref20] Culver S. P.; Squires A. G.; Minafra N.; Armstrong C. W. F.; Krauskopf T.; Böcher F.; Li C.; Morgan B. J.; Zeier W. G. Evidence for a solid-electrolyte inductive effect in the superionic conductor Li_10_Ge_1–*x*_Sn_*x*_P_2_S_12_. J. Am. Chem. Soc. 2020, 142, 21210 10.1021/jacs.0c10735. 33284622 PMC8016198

[ref21] Deiseroth H.-J.; Kong S.-T.; Eckert H.; Vannahme J.; Reiner C.; Zaiß T.; Schlosser M. Li_6_PS_5_X: A class of crystalline Li-rich solids with an unusually high Li^+^ mobility. Angew. Chem. Int. Ed. 2008, 47, 755 10.1002/anie.200703900. 18161703

[ref22] de Klerk N. J. J.; Rosłoń I.; Wagemaker M. Diffusion mechanism of Li argyrodite solid electrolytes for Li-ion batteries and prediction of optimized halogen doping: The effect of Li vacancies, halogens, and halogen disorder. Chem. Mater. 2016, 28, 7955 10.1021/acs.chemmater.6b03630.

[ref23] Kraft M. A.; Culver S. P.; Calderon M.; Böcher F.; Krauskopf T.; Senyshyn A.; Dietrich C.; Zevalkink A.; Janek J.; Zeier W. G. Influence of lattice polarizability on the ionic conductivity in the lithium superionic argyrodites Li_6_PS_5_X (X = Cl, Br, I). J. Am. Chem. Soc. 2017, 139, 10909 10.1021/jacs.7b06327. 28741936

[ref24] Minafra N.; Culver S. P.; Krauskopf T.; Senyshyn A.; Zeier W. G. Effect of Si substitution on the structural and transport properties of superionic Li-argyrodites. J. Mater. Chem. A 2018, 6, 645 10.1039/c7ta08581h.

[ref25] Adeli P.; Bazak J. D.; Park K. H.; Kochetkov I.; Huq A.; Goward G. R.; Nazar L. F. Boosting solid-state diffusivity and conductivity in lithium superionic argyrodites by halide substitution. Angew. Chem. Int. Ed. 2019, 58, 8681 10.1002/anie.201814222. 31041839

[ref26] Rao R. P.; Adams S. Studies of lithium argyrodite solid electrolytes for all-solid-state batteries. Phys. Status Solidi A 2011, 208, 1804 10.1002/pssa.201001117.

[ref27] Rayavarapu P. R.; Sharma N.; Peterson V. K.; Adams S. Variation in structure and Li^+^-ion migration in argyrodite-type Li_6_PS_5_X (X = Cl, Br, I) solid electrolytes. J. Solid State Electrochem. 2012, 16, 1807 10.1007/s10008-011-1572-8.

[ref28] Hanghofer I.; Brinek M.; Eisbacher S. L.; Bitschnau B.; Volck M.; Hennige V.; Hanzu I.; Rettenwander D.; Wilkening H. M. R. Substitutional disorder: structure and ion dynamics of the argyrodites Li_6_PS_5_Cl, Li_6_PS_5_Br and Li_6_PS_5_I. Phys. Chem. Chem. Phys. 2019, 21, 8489 10.1039/c9cp00664h. 30957832

[ref29] Chen H. M.; Maohua C.; Adams S. Stability and ionic mobility in argyrodite-related lithium-ion solid electrolytes. Phys. Chem. Chem. Phys. 2015, 17, 16494 10.1039/c5cp01841b. 26051899

[ref30] Stamminger A. R.; Ziebarth B.; Mrovec M.; Hammerschmidt T.; Drautz R. Ionic conductivity and its dependence on structural disorder in halogenated argyrodites Li_6_PS_5_X (X = Br, Cl, I). Chem. Mater. 2019, 31, 8673 10.1021/acs.chemmater.9b02047.

[ref31] Deng Z.; Zhu Z.; Chu I.-H.; Ong S. P. Data-driven first-principles methods for the study and design of alkali superionic conductors. Chem. Mater. 2016, 29, 281 10.1021/acs.chemmater.6b02648.

[ref32] Gautam A.; Sadowski M.; Prinz N.; Eickhoff H.; Minafra N.; Ghidiu M.; Culver S. P.; Albe K.; Fässler T. F.; Zobel M.; Zeier W. G. Rapid crystallization and kinetic freezing of site-disorder in the lithium superionic argyrodite Li_6_PS_5_Br. Chem. Mater. 2019, 31, 10178 10.1021/acs.chemmater.9b03852.

[ref33] Baktash A.; Reid J. C.; Roman T.; Searles D. J. Diffusion of lithium ions in lithium-argyrodite solid-state electrolytes. npj Comput. Mater. 2020, 6, 162 10.1038/s41524-020-00432-1.

[ref34] Feng X.; Chien P.-H.; Wang Y.; Patel S.; Wang P.; Liu H.; Immediato-Scuotto M.; Hu Y.-Y. Enhanced ion conduction by enforcing structural disorder in Li-deficient argyrodites Li_6–*x*_PS_5–*x*_Cl_1+*x*_. Energy Storage Mater. 2020, 30, 67 10.1016/j.ensm.2020.04.042.

[ref35] von Unterrichter J.; Range K.-J. Ag_8_GeTe_6_, ein vertreter der argyroditfamilie. Z. Naturforsch. 1978, 33, 866 10.1515/znb-1978-0810.

[ref36] Kong S.-T.; Deiseroth H.-J.; Reiner C.; Gün Ö.; Neumann E.; Ritter C.; Zahn D. Lithium argyrodites with phosphorus and arsenic: Order and disorder of lithium atoms, crystal chemistry, and phase transitions. Chem.—Eur. J. 2010, 16, 2198 10.1002/chem.200902470. 20066696

[ref37] Kuhs W. F.; Nitsche R.; Scheunemann K. The argyrodites — a new family of tetrahedrally close-packed structures. Mater. Res. Bull. 1979, 14, 241 10.1016/0025-5408(79)90125-9.

[ref38] Frank F. C.; Kasper J. S. Complex alloy structures regarded as sphere packings. I. definitions and basic principles. Acta Crystallogr. 1958, 11, 184 10.1107/s0365110x58000487.

[ref39] Frank F. C.; Kasper J. S. Complex alloy structures regarded as sphere packings. II. analysis and classification of representative structures. Acta Crystallogr. 1959, 12, 483 10.1107/s0365110x59001499.

[ref40] Bonneau C.; O’Keeffe M. Intermetallic crystal structures as foams. Beyond Frank-Kasper. Inorg. Chem. 2014, 54, 808 10.1021/ic5017966. 25247234

[ref41] Bernges T.; Culver S. P.; Minafra N.; Koerver R.; Zeier W. G. Competing structural influences in the Li superionic conducting argyrodites Li_6_PS_5–*x*_Se_*x*_Br (0 ≤ *x* ≤ 1) upon Se substitution. Inorg. Chem. 2018, 57, 13920 10.1021/acs.inorgchem.8b02443. 30345753

[ref42] Ganapathy S.; Yu C.; van Eck E. R. H.; Wagemaker M. Peeking across grain boundaries in a solid-state ionic conductor. ACS Energy Lett. 2019, 4, 1092 10.1021/acsenergylett.9b00610.

[ref43] Hanghofer I.; Gadermaier B.; Wilkening H. M. R. Fast rotational dynamics in argyrodite-type Li_6_PS_5_X (X: Cl, Br, I) as seen by ^31^P nuclear magnetic relaxation—on cation–anion coupled transport in thiophosphates. Chem. Mater. 2019, 31, 4591 10.1021/acs.chemmater.9b01435.

[ref44] Wang P.; Liu H.; Patel S.; Feng X.; Chien P.-H.; Wang Y.; Hu Y.-Y. Fast ion conduction and its origin in Li_6–*x*_PS_5–*x*_Br_1+*x*_. Chem. Mater. 2020, 32, 3833 10.1021/acs.chemmater.9b05331.

[ref45] Deiseroth H.-J.; Maier J.; Weichert K.; Nickel V.; Kong S.-T.; Reiner C. Li_7_PS_6_ and Li_6_PS_5_X (X: Cl, Br, I): Possible three-dimensional diffusion pathways for lithium ions and temperature dependence of the ionic conductivity by impedance measurements. Z. Anorg. Allg. Chem. 2011, 637, 1287 10.1002/zaac.201100158.

[ref46] Minafra N.; Kraft M. A.; Bernges T.; Li C.; Schlem R.; Morgan B. J.; Zeier W. G. Local charge inhomogeneity and lithium distribution in the superionic argyrodites Li_6_PS_5_X (X = Cl, Br, I). Inorg. Chem. 2020, 59, 11009 10.1021/acs.inorgchem.0c01504. 32673483

[ref47] Schlenker R.; Hansen A.-L.; Senyshyn A.; Zinkevich T.; Knapp M.; Hupfer T.; Ehrenberg H.; Indris S. Structure and diffusion pathways in Li_6_PS_5_Cl argyrodite from neutron diffraction, pair-distribution function analysis and NMR. Chem. Mater. 2020, 32, 8420 10.1021/acs.chemmater.0c02418.

[ref48] Zhou L.; Assoud A.; Zhang Q.; Wu X.; Nazar L. F. A new family of argyrodite thioantimonate lithium superionic conductors. J. Am. Chem. Soc. 2019, 141, 19002 10.1021/jacs.9b08357. 31642663

[ref49] Huang W.; Yoshino K.; Hori S.; Suzuki K.; Yonemura M.; Hirayama M.; Kanno R. Superionic lithium conductor with a cubic argyrodite-type structure in the Li–Al–Si–S system. J. Solid State Chem. 2019, 270, 487 10.1016/j.jssc.2018.12.015.

[ref50] Huang W.; Cheng L.; Hori S.; Suzuki K.; Yonemura M.; Hirayama M.; Kanno R. Ionic conduction mechanism of a lithium superionic argyrodite in the Li-Al-Si-S-O system. Mater. Adv. 2020, 1, 334 10.1039/d0ma00115e.

[ref51] Kresse G.; Furthmüller J. Efficient iterative schemes for ab initio total-energy calculations using a plane-wave basis set. Phys. Rev. B: Condens. Matter Mater. Phys. 1996, 54, 11169 10.1103/physrevb.54.11169. 9984901

[ref52] Kresse G.; Furthmüller J. Efficiency of ab-initio total energy calculations for metals and semiconductors using a plane-wave basis set. Comput. Mater. Sci. 1996, 6, 15 10.1016/0927-0256(96)00008-0. 9984901

[ref53] Perdew J. P.; Ruzsinszky A.; Csonka G.; Vydrov O.; Scuseria G.; Constantin L.; Zhou X.; Burke K. Restoring the density-gradient expansion for exchange in solids and surfaces. Phys. Rev. Lett. 2008, 100, 136406 10.1103/physrevlett.100.136406. 18517979

[ref54] Kresse G.; Joubert D. From ultrasoft pseudopotentials to the projector augmented-wave method. Phys. Rev. B: Condens. Matter Mater. Phys. 1999, 59, 1758 10.1103/physrevb.59.1758.

[ref55] Persson K. Materials Data on Li_6_PS_5_Cl (SG:216) by Materials Project, 2016.

[ref56] Keys A. S.; Hedges L. O.; Garrahan J. P.; Glotzer S. C.; Chandler D. Excitations are localized and relaxation is hierarchical in glass-forming liquids. Phys. Rev. X 2011, 1, 021013 10.1103/physrevx.1.029901.

[ref57] Stillinger F. H.; Weber T. A. Packing structures and transitions in liquids and solids. Science 1984, 225, 983 10.1126/science.225.4666.983. 17783020

[ref58] Heuer A. Exploring the potential energy landscape of glass-forming systems: From inherent structures via metabasins to macroscopic transport. J. Phys. Condens. Matter 2008, 20, 373101 10.1088/0953-8984/20/37/373101. 21694408

[ref59] Morgan B. J. DFT dataset for mechanistic origin of superionic lithium diffusion in anion-disordered Li_6_PS_5_X argyrodites. 2020, https://doi.org/10.15125/BATH-00814 (accessed Feb 09, 2021). 10.1021/acs.chemmater.0c03738 PMC8029578 33840894

[ref60] Morgan B. J. Data analysis for “mechanistic origin of superionic lithium diffusion in anion-disordered Li_6_PS_5_X argyrodites”. 2020, https://doi.org/10.5281/zenodo.4338578 (accessed Feb 09, 2021). 10.1021/acs.chemmater.0c03738 PMC8029578 33840894

[ref61] Hunter J. D. Matplotlib: A 2d graphics environment. Comput. Sci. Eng. 2007, 9, 90 10.1109/mcse.2007.55.

[ref62] Harris C. R.; Millman K. J.; van der Walt S. J.; Gommers R.; Virtanen P.; Cournapeau D.; Wieser E.; Taylor J.; Berg S.; Smith N. J.; Kern R.; Picus M.; Hoyer S.; van Kerkwijk M. H.; Brett M.; Haldane A.; del Río J. F.; Wiebe M.; Peterson P.; Gérard-Marchant P.; Sheppard K.; Reddy T.; Weckesser W.; Abbasi H.; Gohlke C.; Oliphant T. E. Array programming with NumPy. Nature 2020, 585, 357 10.1038/s41586-020-2649-2. 32939066 PMC7759461

[ref63] Ong S. P.; Richards W. D.; Jain A.; Hautier G.; Kocher M.; Cholia S.; Gunter D.; Chevrier V. L.; Persson K. A.; Ceder G. Python materials genomics (pymatgen): A robust, open-source python library for materials analysis. Comput. Mater. Sci. 2013, 68, 314 10.1016/j.commatsci.2012.10.028.

[ref64] Waroquiers D.; George J.; Horton M.; Schenk S.; Persson K. A.; Rignanese G.-M.; Gonze X.; Hautier G. ChemEnv: a fast and robust coordination environment identification tool. Acta Crystallogr., Sect. B: Struct. Sci., Cryst. Eng. Mater. 2020, 76, 683 10.1107/s2052520620007994. PMC7412753 32831287

[ref65] Virtanen P.; Gommers R.; Gommers R.; Oliphant T. E.; Haberland M.; Reddy T.; Cournapeau D.; Burovski E.; Peterson P.; Weckesser W.; Bright J.; van der Walt S. J.; Brett M.; Wilson J.; Millman K. J.; Mayorov N.; Nelson A. R. J.; Jones E.; Kern R.; Larson E.; Carey C. J.; Polat İ.; Feng Y.; Moore E. W.; VanderPlas J.; Laxalde D.; Perktold J.; Cimrman R.; Henriksen I.; Quintero E. A.; Harris C. R.; Archibald A. M.; Ribeiro A. H.; Pedregosa F.; van Mulbregt P. SciPy 1.0: fundamental algorithms for scientific computing in python. Nat. Methods 2020, 17, 261 10.1038/s41592-019-0686-2. 32015543 PMC7056644

[ref66] da Costa-Luis C. O. tqdm: A fast, extensible progress meter for Python and CLI. J. Open Source Software 2019, 4, 1277 10.21105/joss.01277.

[ref67] Morgan B. J. vasppy: A Python suite for manipulating VASP input and output. https://github.com/bjmorgan/vasppy (accessed Feb 09, 20201).

[ref68] Site-analysis codebase. https://github.com/bjmorgan/site-_analysis (accessed Feb 09, 2021).

[ref69] Polyhedral-analysis codebase. https://github.com/bjmorgan/polyhedral-_analysis (accessed Feb 09, 2021).

[ref70] Kinisi codebase. https://github.com/bjmorgan/kinisi (accessed Feb 09, 2021).

[ref71] O’Rourke C.; Morgan B. J. crystal-torture: A crystal tortuosity module. J. Open Source Software 2019, 4, 1306 10.21105/joss.01306.

[ref72] Morgan B. J.; Madden P. A. Absence of a space-charge-derived enhancement of ionic conductivity in β|γ-heterostructured 7H- and 9R-AgI. J. Phys. Condens. Matter 2012, 24, 275303 10.1088/0953-8984/24/27/275303. 22713865

[ref73] Efron B. Bootstrap methods: Another look at the jackknife. Ann. Stat. 1979, 7, 1 10.1214/aos/1176344552.

[ref74] Because the anion substructure is tetrahedrally close-packed, using anion-position-defined tetrahedra to define discrete sites guarantees every lithium ion is assigned to one, and only one, tetrahedral site at each simulation time-step.

[ref75] Yu C.; van Eijck L.; Ganapathy S.; Wagemaker M. Synthesis, structure and electrochemical performance of the argyrodite Li_6_PS_5_Cl solid electrolyte for Li-ion solid state batteries. Electrochim. Acta 2016, 215, 93 10.1016/j.electacta.2016.08.081.

[ref76] We note that the 2 → 2 and 5 → 4 → 5 pathways correspond to the two “intercage” diffusion pathways proposed by Schlenker et al. on the basis of differential bond-valence analysis.^47^

[ref77] Pinsky M.; Avnir D. Continuous symmetry measures. 5. The classical polyhedra. Inorg. Chem. 1998, 37, 5575 10.1021/ic9804925. 11670704

[ref78] Waroquiers D.; Gonze X.; Rignanese G.-M.; Welker-Nieuwoudt C.; Rosowski F.; Göbel M.; Schenk S.; Degelmann P.; André R.; Glaum R.; Hautier G. Statistical analysis of coordination environments in oxides. Chem. Mater. 2017, 29, 8346 10.1021/acs.chemmater.7b02766.

[ref79] For Li_6_PS_5_I with 0 and 100 % site-inversion, 100 and 100 % of the S–Li coordination polyhedra are 6-coordinate, respectively. For Li_6_PS_5_Cl with 0 and 100 % site-inversion, 99.98 and 99.57% of S–Li coordination polyhedra are 6-coordinate, respectively.

[ref80] For the 0 and 100% site-inverted Li_6_PS_5_I systems, we observe no exchange of lithium between coordination polyhedra during the 70 ps simulations. For Li_6_PS_5_Cl with 0 and 100% site-inversion, we observe Li-exchange between coordination polyhedra with frequencies of 0.002/polyhedron/ps and 0.019/polyhedron/ps, respectively.

[ref81] Casanova D.; Cirera J.; Llunell M.; Alemany P.; Avnir D.; Alvarez S. Minimal distortion pathways in polyhedral rearrangements. J. Am. Chem. Soc. 2004, 126, 1755 10.1021/ja036479n. 14871107

[ref82] Morgan B. J. Lattice-geometry effects in garnet solid electrolytes: a lattice-gas Monte Carlo simulation study. R. Soc. Open Sci. 2017, 4, 170824 10.1098/rsos.170824. 29291073 PMC5717647

[ref83] Van der Ven A.; Bhattacharya J.; Belak A. A. Understanding Li diffusion in Li-intercalation compounds. Acc. Chem. Res. 2013, 46, 1216 10.1021/ar200329r. 22584006

[ref84] Catlow C. Static lattice simulation of structure and transport in superionic conductors. Solid State Ionics 1983, 8, 89 10.1016/0167-2738(83)90069-3.

[ref85] Ohno S.; Helm B.; Fuchs T.; Dewald G.; Kraft M. A.; Culver S. P.; Senyshyn A.; Zeier W. G. Further evidence for energy landscape flattening in the superionic argyrodites Li_6+*x*_P_1–*x*_M_*x*_S_5_I (M = Si, Ge, Sn). Chem. Mater. 2019, 31, 4936 10.1021/acs.chemmater.9b01857.

[ref86] Annamareddy A.; Eapen J. Low dimensional string-like relaxation underpins superionic conduction in fluorites and related structures. Sci. Rep. 2017, 7, 44149 10.1038/srep44149. 28344314 PMC5366808

[ref87] Catlow C. R. A. Atomistic mechanisms of ionic transport in fast-ion conductors. Faraday Trans. 1990, 86, 1167 10.1039/ft9908601167.

[ref88] Catlow C. R. A. Defect processes and migration mechanisms in solid state ionics. Mater. Sci. Eng., B 1992, 12, 375 10.1016/0921-5107(92)90009-x.

[ref89] Mohn C. E.; Krynski M. Collective diffusion within the superionic regime of Bi_2_O_3_. Phys. Rev. B 2020, 101, 104309 10.1103/physrevb.101.104309.

[ref90] Salanne M.; Marrocchelli D.; Watson G. W. Cooperative mechanism for the diffusion of Li^+^ ions in LiMgSO_4_F. J. Phys. Chem. C 2012, 116, 18618 10.1021/jp304767d.

[ref91] Zhang B.; Yang L.; Wang L.-W.; Pan F. Cooperative transport enabling fast Li-ion diffusion in thio-LISICON Li_10_SiP_2_S_12_ solid electrolyte. Nano Energy 2019, 62, 844 10.1016/j.nanoen.2019.05.085.

[ref92] Zhang H.; Wang X.; Chremos A.; Douglas J. F. Superionic UO_2_: A model anharmonic crystalline material. J. Chem. Phys. 2019, 150, 174506 10.1063/1.5091042. 31067868

[ref93] He X.; Zhu Y.; Mo Y. Origin of fast ion diffusion in super-ionic conductors. Nat. Commun. 2017, 8, 15893 10.1038/ncomms15893. 28635958 PMC5482052

[ref94] Xu M.; Ding J.; Ma E. One-dimensional stringlike cooperative migration of lithium ions in an ultrafast ionic conductor. Appl. Phys. Lett. 2012, 101, 031901 10.1063/1.4737397.

[ref95] Yokota I. On the deviation from the Einstein relation observed for diffusion of Ag ions in α-Ag_2_S and others. J. Phys. Soc. Jpn. 1966, 21, 420 10.1143/jpsj.21.420.

[ref96] Zendejas M. A.; Thomas J. O. Conduction mechanisms in solid electrolytes: Na^+^ beta-alumina. Phys. Scr. 1990, T33, 235 10.1088/0031-8949/1990/t33/045.

[ref97] Donati C.; Douglas J. F.; Kob W.; Plimpton S. J.; Poole P. H.; Glotzer S. C. Stringlike cooperative motion in a supercooled liquid. Phys. Rev. Lett. 1998, 80, 2338 10.1103/physrevlett.80.2338.

[ref98] Morgan B. J.; Madden P. A. Relationships between atomic diffusion mechanisms and ensemble transport coefficients in crystalline polymorphs. Phys. Rev. Lett. 2014, 112, 145901 10.1103/physrevlett.112.145901. 24765989

[ref99] Glotzer S. C. Spatially heterogeneous dynamics in liquids: Insights from simulation. J. Non-Cryst. Solids 2000, 274, 342 10.1016/s0022-3093(00)00225-8.

[ref100] Zhang H.; Zhong C.; Douglas J. F.; Wang X.; Cao Q.; Zhang D.; Jiang J.-Z. Role of string-like collective atomic motion on diffusion and structural relaxation in glass forming Cu-Zr alloys. J. Chem. Phys. 2015, 142, 164506 10.1063/1.4918807. 25933773

[ref101] Wang Y.-J.; Du J.-P.; Shinzato S.; Dai L.-H.; Ogata S. A free energy landscape perspective on the nature of collective diffusion in amorphous solids. Acta Mater. 2018, 157, 165 10.1016/j.actamat.2018.07.029.

[ref102] Zhang Z.; Zhang J.; Jia H.; Peng L.; An T.; Xie J. Enhancing ionic conductivity of solid electrolyte by lithium substitution in halogenated Li-argyrodite. J. Power Sources 2020, 450, 227601 10.1016/j.jpowsour.2019.227601.

[ref103] Yu C.; Li Y.; Willans M.; Zhao Y.; Adair K. R.; Zhao F.; Li W.; Deng S.; Liang J.; Banis M. N.; Li R.; Huang H.; Zhang L.; Yang R.; Lu S.; Huang Y.; Sun X. Superionic conductivity in lithium argyrodite solid-state electrolyte by controlled Cl-doping. Nano Energy 2020, 69, 104396 10.1016/j.nanoen.2019.104396.

[ref104] Kozinsky B. Transport in frustrated and disordered solid electrolytes. Handbook of Materials Modeling; Springer International Publishing, 2018; pp 1–20.

[ref105] Hayes W. Superionic conductors, Contemp. Phys 1978, 19, 469 10.1080/00107517808210895.

[ref106] Villa M.; Bjorkstam J. The role of disorder in superionic conductors. Solid State Ionics 1980, 1, 481 10.1016/0167-2738(80)90044-2.

[ref107] Keen D. A. Disordering phenomena in superionic conductors. J. Phys.: Condens. Matter 2002, 14, R819 10.1088/0953-8984/14/32/201.

[ref108] Hull S. Superionics: Crystal structures and conduction processes. Rep. Prog. Phys. 2004, 67, 1233 10.1088/0034-4885/67/7/r05.

[ref109] Dathar G. K. P.; Balachandran J.; Kent P. R. C.; Rondinone A. J.; Ganesh P. Li-ion site disorder driven superionic conductivity in solid electrolytes: a first-principles investigation of β-Li_3_PS_4_. J. Mater. Chem. A 2017, 5, 1153 10.1039/C6TA07713G.

[ref110] Di Stefano D.; Miglio A.; Robeyns K.; Filinchuk Y.; Lechartier M.; Senyshyn A.; Ishida H.; Spannenberger S.; Prutsch D.; Lunghammer S.; Rettenwander D.; Wilkening M.; Roling B.; Kato Y.; Hautier G. Superionic diffusion through frustrated energy landscape. Chem 2019, 5, 2450 10.1016/j.chempr.2019.07.001.

[ref111] Kweon K. E.; Varley J. B.; Shea P.; Adelstein N.; Mehta P.; Heo T. W.; Udovic T. J.; Stavila V.; Wood B. C. Structural, chemical, and dynamical frustration: Origins of superionic conductivity in closo-borate solid electrolytes. Chem. Mater. 2017, 29, 9142 10.1021/acs.chemmater.7b02902.

[ref112] Jørgensen M.; Shea P. T.; Tomich A. W.; Varley J. B.; Bercx M.; Lovera S.; Černý R.; Zhou W.; Udovic T. J.; Lavallo V.; Jensen T. R.; Wood B. C.; Stavila V. Understanding superionic conductivity in lithium and sodium salts of weakly coordinating closo-hexahalocarbaborate anions. Chem. Mater. 2020, 32, 1475 10.1021/acs.chemmater.9b04383.

[ref113] Leube B. T.; Inglis K. K.; Carrington E. J.; Sharp P. M.; Shin J. F.; Neale A. R.; Manning T. D.; Pitcher M. J.; Hardwick L. J.; Dyer M. S.; Blanc F.; Claridge J. B.; Rosseinsky M. J. Lithium transport in Li_4.4_M_0.4_M_0.6_^′^S_4_(M = Al^3+^, Ga^3+^, and M′ = Ge^4+^, Sn^4+^): Combined crystallographic, conductivity, solid state NMR, and computational studies. Chem. Mater. 2018, 30, 7183 10.1021/acs.chemmater.8b03175.

[ref114] Düvel A.; Heitjans P.; Fedorov P.; Scholz G.; Cibin G.; Chadwick A. V.; Pickup D. M.; Ramos S.; Sayle L. W. L.; Sayle E. K. L.; Sayle T. X. T.; Sayle D. C. Is geometric frustration-induced disorder a recipe for high ionic conductivity?. J. Am. Chem. Soc. 2017, 139, 5842 10.1021/jacs.7b00502. 28362104

[ref115] Breuer S.; Wilkening M. Mismatch in cation size causes rapid anion dynamics in solid electrolytes: the role of the arrhenius pre-factor. Dalton Trans. 2018, 47, 4105 10.1039/c7dt04487a. 29465125

[ref116] Deng Y.; Eames C.; Fleutot B.; David R.; Chotard J.-N.; Suard E.; Masquelier C.; Islam M. S. Enhancing the lithium ion conductivity in lithium superionic conductor LISICON solid electrolytes through a mixed polyanion effect. ACS Appl. Mater. Interfaces 2017, 9, 7050 10.1021/acsami.6b14402. 28128548

[ref117] Marple M. A. T.; Aitken B. G.; Kim S.; Sen S. Observation of a phonon softening effect on Li ion conduction in mixed-anion chalcogenide glasses. Chem. Mater. 2018, 30, 5896 10.1021/acs.chemmater.8b01830.

[ref118] Zhang Y.; Zhao Y.; Chen C. Ab initio study of the stabilities of and mechanism of superionic transport in lithium-rich antiperovskites. Phys. Rev. B: Condens. Matter Mater. Phys. 2013, 87, 134303 10.1103/physrevb.87.134303.

[ref119] Dyre J. C.; Maass P.; Roling B.; Sidebottom D. L. Fundamental questions relating to ion conduction in disordered solids. Rep. Prog. Phys. 2009, 72, 046501 10.1088/0034-4885/72/4/046501.

