# Supplementary material for: Mechanistic Origin of Superionic Lithium Diffusion in Anion-Disordered Li6PS5X Argyrodites
Source: Chem Mater. 2021 Mar 3;33(6):2004–18. doi: 10.1021/acs.chemmater.0c03738 (PMC8029578; doi:10.1021/acs.chemmater.0c03738)
Supplement: Supplementary file 1 — cm0c03738_si_001.pdf [file cm0c03738_si_001.pdf]

Supporting Information for "Mechanistic Origin of Superionic Lithium Diffusion in Anion-Disordered Li_6PS_5X Argyrodites"

Benjamin J. Morgan[1,2]
[1] Department of Chemistry, University of Bath, Claverton Down BA2 7AY, United Kingdom
[2] The Faraday Institution, Quad One, Harwell Science and Innovation Campus, Didcot OX11 0RA, United Kingdom*
(Dated: February 13, 2021)

S1. MECHANISTIC ANALYSIS FROM INHERENT STRUCTURES VERSUS UNPROCESSED TRAJECTORIES

Comparison of unprocessed versus inherent-structure trajectories

Our focus in this study is on the underlying diffusion mechanisms operating in each molecular dynamics simulation. To help identify these we post-process each simulation trajectory to obtain a set of "inherent structures" that capture the key diffusive motions of the lithium ions [1–3]. Each inherent structure is generated by taking a "snapshot" single configuration from the relevant molecular dynamics trajectory and performing a conjugate-gradient geometry optimisation, which relaxes the structure into a local potential energy minimum. By repeating this process for a sequence of configurations sampled at fixed time-intervals we obtain an "inherent-structure trajectory" that can then be analysed alongside the original unprocessed simulation trajectory.

These inherent-structure trajectories are useful because we are interested in identifying *non-trivial* displacements of lithium ions, i.e. those displacements that contribute to net lithium diffusion. These are different to vibrational motions, which produce short-lived lithium displacements that do not contribute to meaningful lithium diffusion. For a given starting configuration, performing a geometry optimisation to obtain the corresponding inherent structure has the effect of "quenching out" much of this high-frequency thermal motion, providing a clearer description of the underlying diffusion mechanisms.

Figure S1 shows the unprocessed x, y, z coordinates for a single lithium ion from the 50 % site-inverted Li_6PS_5Cl siulation, and the corresponding inherent structure coordinates. The inherent structure trajectory tracks the unprocessed trajectory, but filters out much of the short-timescale short-ranged vibrational motion. The larger scale diffusion motion that describes the movement of the lithium ion through the host framework, however, is preserved.

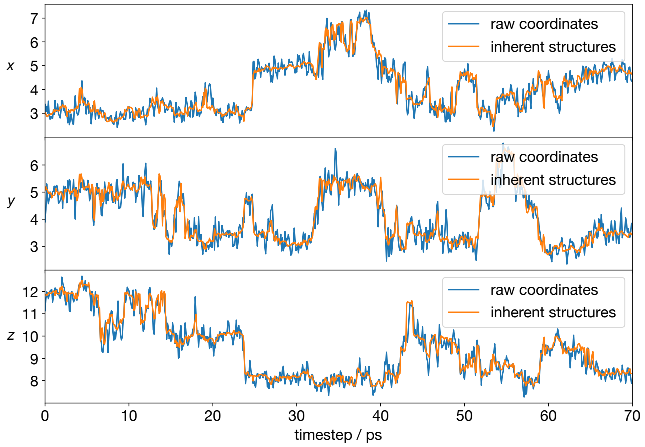

FIG. S1. x, y, z coordinates of a single lithium ion during the 50 % site-inverted Li_6PS_5Cl simulation, from both the unprocessed simulation trajectory ("raw coordinates") and from the corresponding inherent structures. Figure adapted from Ref. [4] under a CC BY-SA 4.0 license https://creativecommons.org/licenses/by-sa/4.0.

Tetrahedral-site–projections from unprocessed versus inherent-structure trajectories

To help understand the different mechanisms of lithium diffusion in our simulations, part of our analysis of the simulation trajectories consists of an analysis of lithium positions and dynamics in terms of occupation of the set of tetrahedral holes formed by the close-packed S/X-anion substructure. Occupation of a specific tetrahedron broadly corresponds to a lithium ion being assigned as occupying the corresponding crystallographic site, and transitions between adjacent tetrahedra give a discretised "site-to-site" description of the lithium diffusion process.

Because the calculation of each inherent structure involves performing a geometry optimisation to relax the structure into a local potential energy minimum, the structures of any given molecular dynamics configuration and its corresponding inherent structure differ. For any single molecular dynamics timestep, this can lead to individual lithium ions being assigned to different sites depending on whether the coordinates from the raw trajectory or from the corresponding inherent structure are used when calculating the tetrahedral-site occupations.

This behaviour is illustrated in Figure S2, which shows

* b.j.morgan@bath.ac.uk

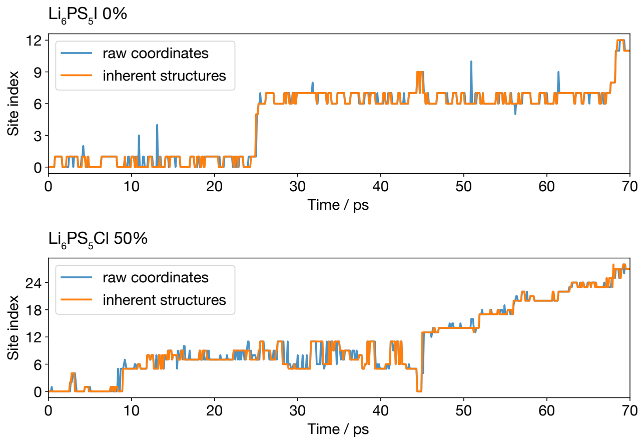

FIG. S2. Site-indices for the tetrahedral sites deemed "occupied" by a single lithium ion during the 0 % Li_6PS_5I (upper panel) and 50 % Li_6PS_5Cl (lower panel simulations, assigned from the unprocessed simulation trajectories and from the corresponding inherent-structure trajectories. The site numbering is arbitrary and in each case is assigned based on the order of sites occupied in the unprocessed trajectory. Figure adapted from Ref. [4] under a CC BY-SA 4.0 license https://creativecommons.org/licenses/by-sa/4.0.

the indices of the sites assigned as "occupied" at each timestep for a single lithium ion from the 0 % site-inverted Li_6PS_5I simulation (upper panel) and from the 50 % site-inverted Li_6PS_5Cl simulation (lower panel). Both site-occupation trajectories follow the same general sequence of sites, and describe the same underlying diffusion behaviour. The site-occupation trajectory obtained from the unprocessed simulation coordinates, however, contains transient site–to–site transitions that are absent from the inherent structure trajectory. These additional transitions correspond to short-lived vibrational motions; in nearly every case the transition to a new site does not persist beyond a single analysis frame. Working with the inherent structure trajectory, therefore, has the benefit of filtering out these non-diffusive "vibrational" transitions between sites, while preserving the transitions that describe the time-evolution of the lithium-ion configuration that underpins the key lithium-diffusion processes.

DATA AVAILABILITY

A dataset containing inputs and outputs for all DFT calculations supporting this study is available under the CC-BY-4.0 licence from the University of Bath Research Data Archive [5]. All code used to analyse the simulation trajectories and to generate the corresponding figures is available as a series of Jupyter notebooks under the MIT licence as Ref. [4].

[1] A. S. Keys, L. O. Hedges, J. P. Garrahan, S. C. Glotzer, and D. Chandler, Excitations are localized and relaxation is hierarchical in glass-forming liquids, Phys. Rev. X **1**, 021013 (2011).

[2] F. H. Stillinger and T. A. Weber, Packing structures and transitions in liquids and solids, Science **225**, 983 (1984).

[3] A. Heuer, Exploring the potential energy landscape of glass-forming systems: From inherent structures via metabasins to macroscopic transport, J. Phys-Condens. Mat. **20**, 373101 (2008).

[4] B. J. Morgan, Data analysis for "mechanistic origin of superionic lithium diffusion in anion-disordered Li_6PS_5X argyrodites" (2020), `https://doi.org/10.5281/zenodo.4338578` (accessed 2021-02-09).

[5] B. J. Morgan, DFT dataset for mechanistic origin of superionic lithium diffusion in anion-disordered Li_6PS_5X argyrodites (2020), `https://researchdata.bath.ac.uk/id/eprint/814` (accessed 2021-02-09).
